# Targeting macrophage M1 polarization suppression through PCAF inhibition alleviates autoimmune arthritis via synergistic NF-κB and H3K9Ac blockade

**DOI:** 10.1186/s12951-023-02012-z

**Published:** 2023-08-19

**Authors:** Jinteng Li, Feng Ye, Xiaojun Xu, Peitao Xu, Peng Wang, Guan Zheng, Guiwen Ye, Wenhui Yu, Zepeng Su, Jiajie Lin, Yunshu Che, Zhidong Liu, Pei Feng, Qian Cao, Dateng Li, Zhongyu Xie, Yanfeng Wu, Huiyong Shen

**Affiliations:** 1 Department of Orthopedics, The Eighth Affiliated Hospital of Sun Yat-sen University, 518003 Shenzhen, PR China; 2 Center for Biotherapy, The Eighth Affiliated Hospital of Sun Yat-sen University, 518003 Shenzhen, PR China; 3 121 Westmoreland Ave, 10606 White Plains, NY USA; 4 Shenzhen Key Laboratory of Ankylosing Spondylitis, 518003 Shenzhen, PR China; 5 Guangdong Orthopedic Clinical Research Center, 518003 Shenzhen, PR China

**Keywords:** PCAF, M1 polarization, Macrophage, Autoimmune arthritis, Dextran sulfate-based nanoparticles (DSNPs)

## Abstract

Sustained inflammatory invasion leads to joint damage and progressive disability in several autoimmune rheumatic diseases. In recent decades, targeting M1 macrophage polarization has been suggested as a promising therapeutic strategy for autoimmune arthritis. P300/CBP-associated factor (PCAF) is a histone acetyltransferase (HAT) that exhibits a strong positive relationship with the proinflammatory microenvironment. However, whether PCAF mediates M1 macrophage polarization remains poorly studied, and whether targeting PCAF can protect against autoimmune arthritis in vivo remains unclear. Commonly used drugs can cause serious side effects in patients because of their extensive and nonspecific distribution in the human body. One strategy for overcoming this challenge is to develop drug nanocarriers that target the drug to desirable regions and reduce the fraction of drug that reaches undesirable targets. In this study, we demonstrated that PCAF inhibition could effectively inhibit M1 polarization and alleviate arthritis in mice with collagen-induced arthritis (CIA) via synergistic NF-κB and H3K9Ac blockade. We further designed dextran sulfate (DS)-based nanoparticles (DSNPs) carrying garcinol (a PCAF inhibitor) to specifically target M1 macrophages in inflamed joints of the CIA mouse model via SR-A–SR-A ligand interactions. Compared to free garcinol, garcinol-loaded DSNPs selectively targeted M1 macrophages in inflamed joints and significantly improved therapeutic efficacy in vivo. In summary, our study indicates that targeted PCAF inhibition with nanoparticles might be a promising strategy for treating autoimmune arthritis via M1 macrophage polarization inhibition.

**Supplementary Information:**

The online version contains supplementary material available at 10.1186/s12951-023-02012-z.

## Introduction

Macrophages are derived from monocytes, which act as essential guardians of the innate immune system [1]. Macrophages are typically divided into the proinflammatory M1 type and anti-inflammatory M2 type of macrophages [1]. In multiple inflammatory contexts, M1 macrophage activation initiates and maintains a proinflammatory microenvironment, while polarization from the proinflammatory M1 phenotype into the anti-inflammatory M2 phenotype participates in resolving the inflammatory process [2, 3]. Autoimmune rheumatic diseases are a large group of diseases that are characterized by continuous immune activation without overt infection [4, 5]. Joints are commonly susceptible to several autoimmune rheumatic diseases [6, 7]. Sustained inflammatory invasion leads to joint damage and progressive disability [6, 7]. In recent decades, targeting M1 macrophage polarization inhibition has been suggested as a promising therapeutic strategy for autoimmune arthritis [8, 9]. Thus, further study of the M1 macrophage polarization mechanism to identify more potential therapeutic targets is of great clinical importance.

P300/CBP-associated factor (PCAF) is a histone acetyltransferase (HAT) that exhibits a strong positive relationship with the proinflammatory microenvironment [10–12]. PCAF has been proven to be overexpressed under several inflammatory conditions [10–12]. PCAF overexpression was further shown to mediate both histone acetylation and nonhistone protein acetylation to induce the generation of proinflammatory molecules or cytokines [11, 13, 14]. Moreover, PCAF can acetylate p65 to control NF-κB signal activation [15, 16]. NF-κB signal activation is pivotal in both M1 macrophage polarization and the secretion of several proinflammatory cytokines, including TNF-α, IL-6, and IL-1β [17–19]. Accordingly, PCAF inhibition has been proven to sufficiently reduce M1 macrophage-mediated inflammation in vitro [20, 21]. However, the specific mechanism by which PCAF mediates M1 macrophage polarization remains poorly studied, and whether targeting PCAF can protect against M1 macrophage polarization and autoimmune arthritis in vivo remains unclear and warrants further exploration.

Multiple types of drugs, including NSAIDs, DMARDs and biologics, have been widely used both alone and in combination to treat autoimmune rheumatic diseases [22–24]. However, these commonly used drugs, even biologics, still often cause serious side effects in patients because of their extensive and nonspecific distribution in the human body. One strategy for overcoming this challenge is to develop nanocarriers for drugs that target inflamed regions and reduce the fraction of drug that reaches undesirable targets [25, 26]. Specific receptor‒ligand interactions are commonly exploited to improve nanocarrier-specific targeting ability [27, 28]. Scavenger receptor class A (SR-A) is specifically overexpressed by activated macrophages [29, 30]. Thus, using nanocarriers with the SR-A ligand packaged with drugs is a rational and promising strategy to increase drug targeting to activated M1 macrophages to relieve inflammation and to decrease the side effects of drugs [29, 30].

The collagen-induced arthritis (CIA) mouse model is one of the most commonly used animal disease models to study autoimmune arthritis [31, 32]. M1 macrophage activation also plays an essential role in the initiation and maintenance of arthritis in the CIA mouse model [32, 33]. In this study, we demonstrated that PCAF was overexpressed to induce M1 polarization in inflamed joints in the CIA model. PCAF KO mice showed less M1 macrophage polarization and significantly higher resistance to CIA. Additionally, administration of the systemic PCAF inhibitor garcinol effectively reduced M1 macrophage polarization and arthritis severity in inflamed joints in the wild-type CIA mouse model. Further mechanistic studies indicated that PCAF acetylated both NF-κB subunit p65 and histone H3 at the ninth lysine (H3K9). PCAF-mediated p65 acetylation and H3K9ac modification synergistically activated the transcription of M1 macrophage polarization-related molecules and inflammatory cytokines to boost inflammation progression. Finally, we designed DS (DS)-based nanoparticles (DSNPs) carrying garcinol to specifically target M1 macrophages in inflamed joints in the CIA mouse model via SR-A–SR-A ligand interactions. Compared to free garcinol, garcinol-loaded DSNPs selectively targeted M1 macrophages in inflamed joints and significantly improved therapeutic efficacy in vivo. In summary, our study indicated that targeted PCAF inhibition with nanoparticle carriers might be a promising strategy for treating autoimmune arthritis via inhibition of M1 macrophage polarization.

**Scheme 1:**
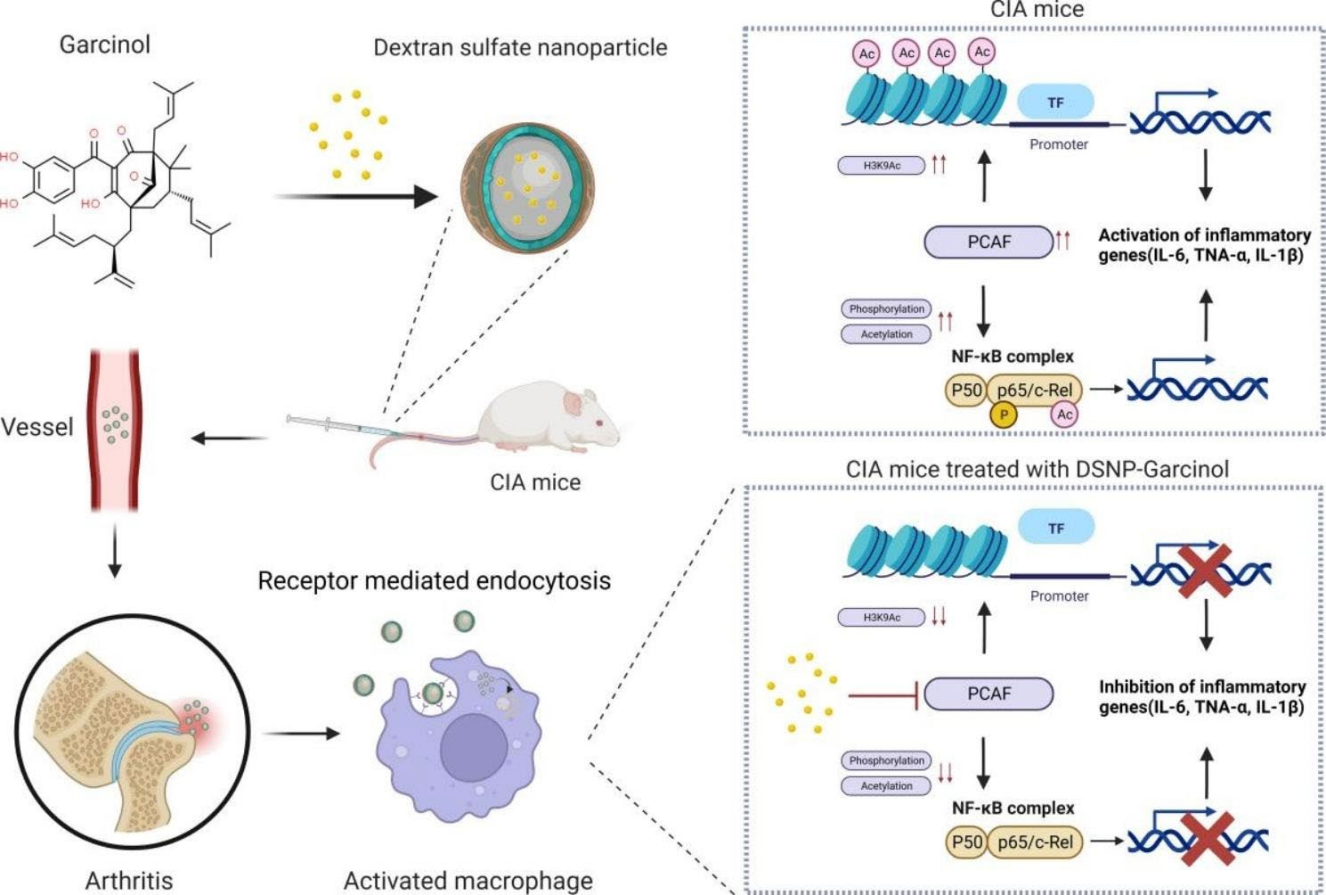
Schematic illustration of the DSNP-mediated improvement of the anti-inflammatory effect of garcinol in a mouse CIA model. DSNPs improved the anti-inflammatory effect of garcinol through several mechanisms: (1) garcinol was effectively loaded into the DSNPs; (2) the DSNPs were selectively taken up by activated macrophages via scavenger receptor class A-mediated endocytosis and effectively accumulated in inflamed joints; and (3) DSNPs enabled the release of garcinol at the intracellular regions of the inflamed site, thereby enhancing the anti-arthritic effect and avoiding the side effects of garcinol. The graphical abstract was created with BioRender.com

## Results and discussion

### PCAF expression was significantly elevated in RA patients and CIA mouse specimens

**Fig. 1:**
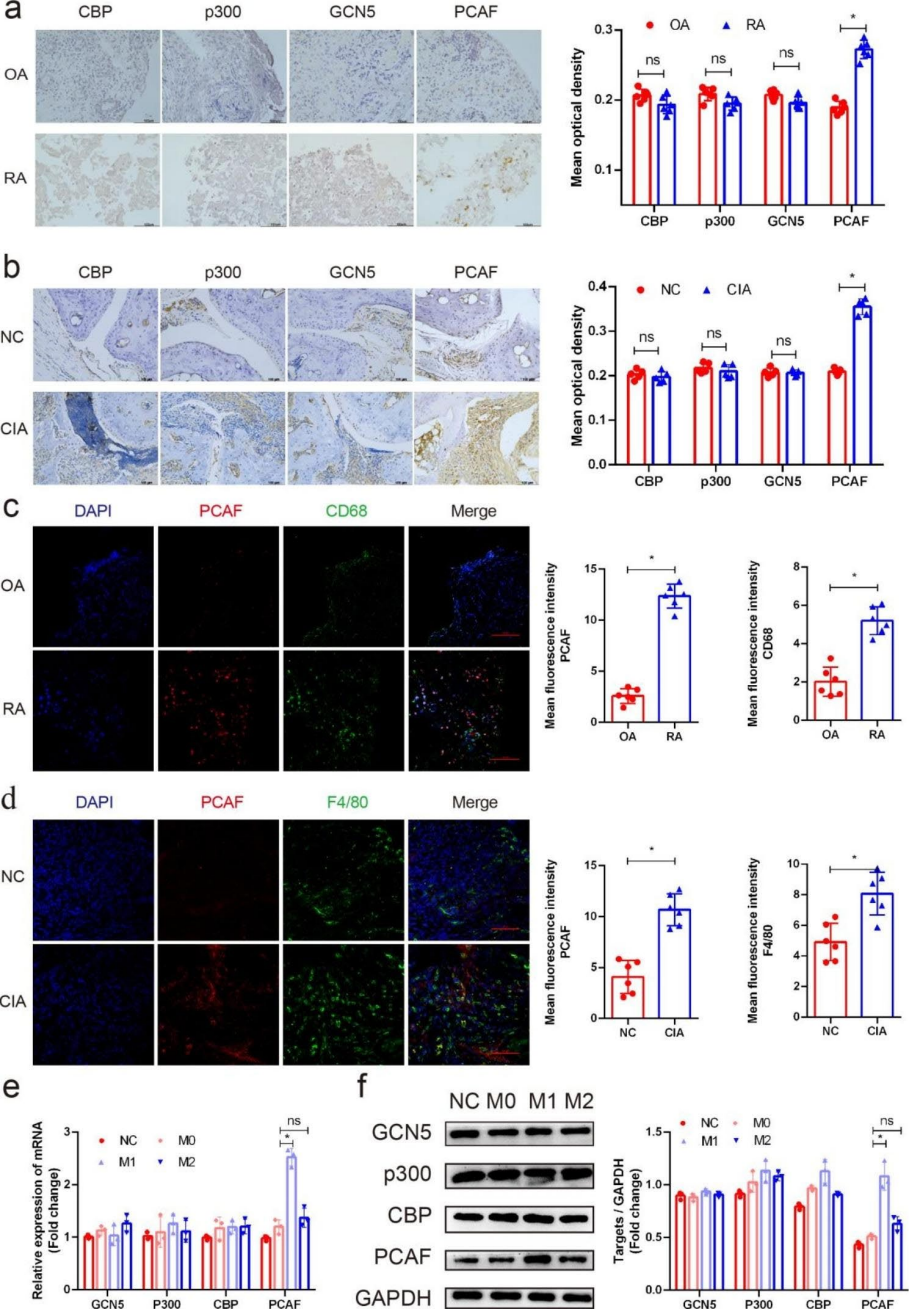
PCAF expression was significantly elevated in RA patients and CIA mice. (**a**) The expression of CBP, p300, GCN5 and PCAF in the synovium of OA and RA patients was detected by IHC. Semiquantification of the mean optical density of CBP, p300, GCN5 and PCAF determined by IHC (n = 6). (**b**) The expression of CBP, p300, GCN5 and PCAF in the synovium of control and CIA mice was detected by IHC. Semiquantification of the mean optical density of CBP, p300, GCN5 and PCAF determined by IHC (n = 5). (**c**) The expression of PCAF in synovial macrophages of OA and RA patients were detect by IF (CD68 green, PCAF red). Semiquantification of the mean fluorescence intensity of PCAF and CD68 determined by IF (n = 6). (**d**) The expression of PCAF in synovial macrophages of control and CIA mice were detect by IF (F4/80 green, PCAF red). Semiquantification of the mean fluorescence intensity of PCAF and F4/80 determined by IF (n = 5). (**e**) The mRNA levels of CBP, p300, GCN5 and PCAF during the polarization of macrophages were determined by qRT‒PCR (n = 3). (**f**) The protein levels of CBP, p300, GCN5 and PCAF during the M1 polarization of macrophages were determined by western blotting (n = 3). ns indicates *P* > 0.05, and * indicates *P* < 0.05

We first explored the expression of PCAF in RA patient synovium and CIA mouse ankle specimens. The results demonstrated that the expression of PCAF was significantly increased in the RA group compared with the OA control group, while the expression of its close “collaborator” GCN5, p300 and CBP did not differ significantly (Fig. 1a). IHC analysis of the inflammatory ankle site of the CIA mice also showed that the expression of PCAF was significantly increased compared with that in the control group, but the expression of P300, CBP and GCN5 was not significantly different (Fig. 1b). Moreover, as shown by immunofluorescence (IF) analysis, the expression of PCAF in macrophages in the synovium of RA patients (Fig. 1c) and inflammatory sites of CIA mice (Fig. 1d) was significantly elevated compared with that in the respective control groups. We further explored the expression of PCAF during the polarization of M1 and M2 macrophages. The mRNA and protein levels of PCAF were significantly upregulated during the differentiation of M1 macrophages, but this change was not observed during the differentiation of M2 macrophages (Fig. 1e, f). These results suggested that PCAF may play an important role in the development of autoimmune arthritis mediated by M1 macrophages.

### PCAF inhibition inhibited M1 polarization in vitro

**Fig. 2:**
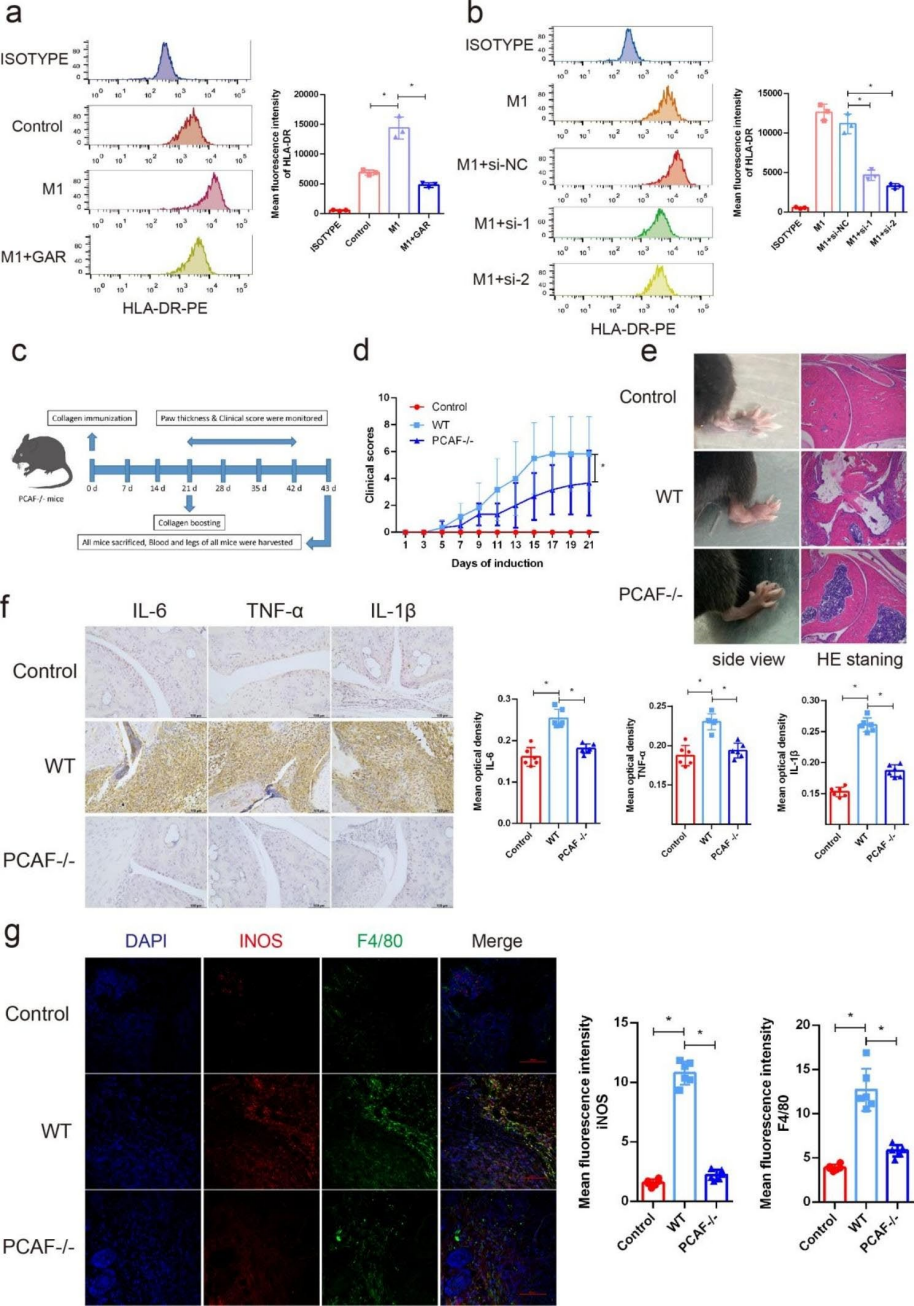
M1 polarization and arthritis were significantly alleviated via PCAF inhibition in vitro and in vivo. (**a**) The MFI of HLA-DR was detected in CD68-positive cells via flow cytometric analysis (n = 3). (**b**) The MFI of HLA-DR was detected in CD68-positive cells via flow cytometric analysis in siRNA assays (n = 3). (**c**) Schematic of CIA in PCAF-/- mice. (**d**) Arthritis severity was determined using a visual arthritis scoring system in WT and PCAF-/- mice (n = 6). (**e**) The general degree of ankle swelling in WT and PCAF-/- mice after CIA induction and corresponding H&E staining of ankle joints excised from the mice are shown. (f) The expression of IL-6, TNF-α and IL-1β in WT and PCAF-/- mice after CIA induction was detected by IHC. Semiquantification of the mean optical density of IL-6, TNF-α and IL-1β determined by IHC (n = 6). (**g**) The expression of iNOS in synovial macrophages of control and CIA mice were detect by IF (F4/80 green, iNOS red). Semiquantification of the mean fluorescence intensity of iNOS and F4/80 determined by IF (n = 6). ns indicates *P* > 0.05, and * indicates *P* < 0.05

The natural compound garcinol, a PCAF inhibitor, was used to further investigate the effect of PCAF on the M1 polarization of human macrophages in vitro. Garcinol significantly decreased the mean fluorescence intensity (MFI) of HLA-DR in CD68-positive cells, indicating inhibition of the polarization of M1 macrophages (Fig. 2a). To further clarify the role of PCAF during the macrophage polarization process, we designed specific siRNAs targeting PCAF to knock down PCAF in macrophages. The polarization of M1 macrophages was also significantly weakened after knockdown of PCAF (Fig. 2b). However, during the polarization of M2 macrophages, neither the PCAF inhibitor (Supplementary Fig. 1a) nor knockdown by siRNA (Supplementary Fig. 1b) had a significant effect on the polarization of M2 macrophages.

### M1 polarization and arthritis were significantly alleviated via PCAF inhibition in vivo

Moreover, to further investigate the role of PCAF in vivo, PCAF gene knockout mice were generated, and CIA was induced (Fig. 2c). DNA gel electrophoresis confirmed that the mice we obtained were PCAF knockout mice (Supplementary Fig. 2). After CIA induction, surprisingly, compared with wild-type mice, PCAF knockout mice had significantly lower joint scores (Fig. 2d) and milder arthritis symptoms (Fig. 2e). Twenty-three days after boost immunization, the mice were sacrificed, and paraffin sections were taken from the hind paws for HE and IHC staining. The results of HE staining showed that PCAF knockout mice had significantly less joint inflammation than wild-type mice (Fig. 2e). In addition, the results of IHC staining suggested that in PCAF knockout mice, the expression of the classic inflammatory cytokines TNF-α, IL-6, and IL-1β was significantly reduced compared with that in wild-type mice (Fig. 2f). Semiquantification of the mean optical density values from IHC analysis also showed significant differences (Fig. 2f). Furthermore, IF indicated that the polarization of M1 macrophages was significantly decreased in PCAF knockout mice compared to wild-type mice (Fig. 2g).

**Fig. 3:**
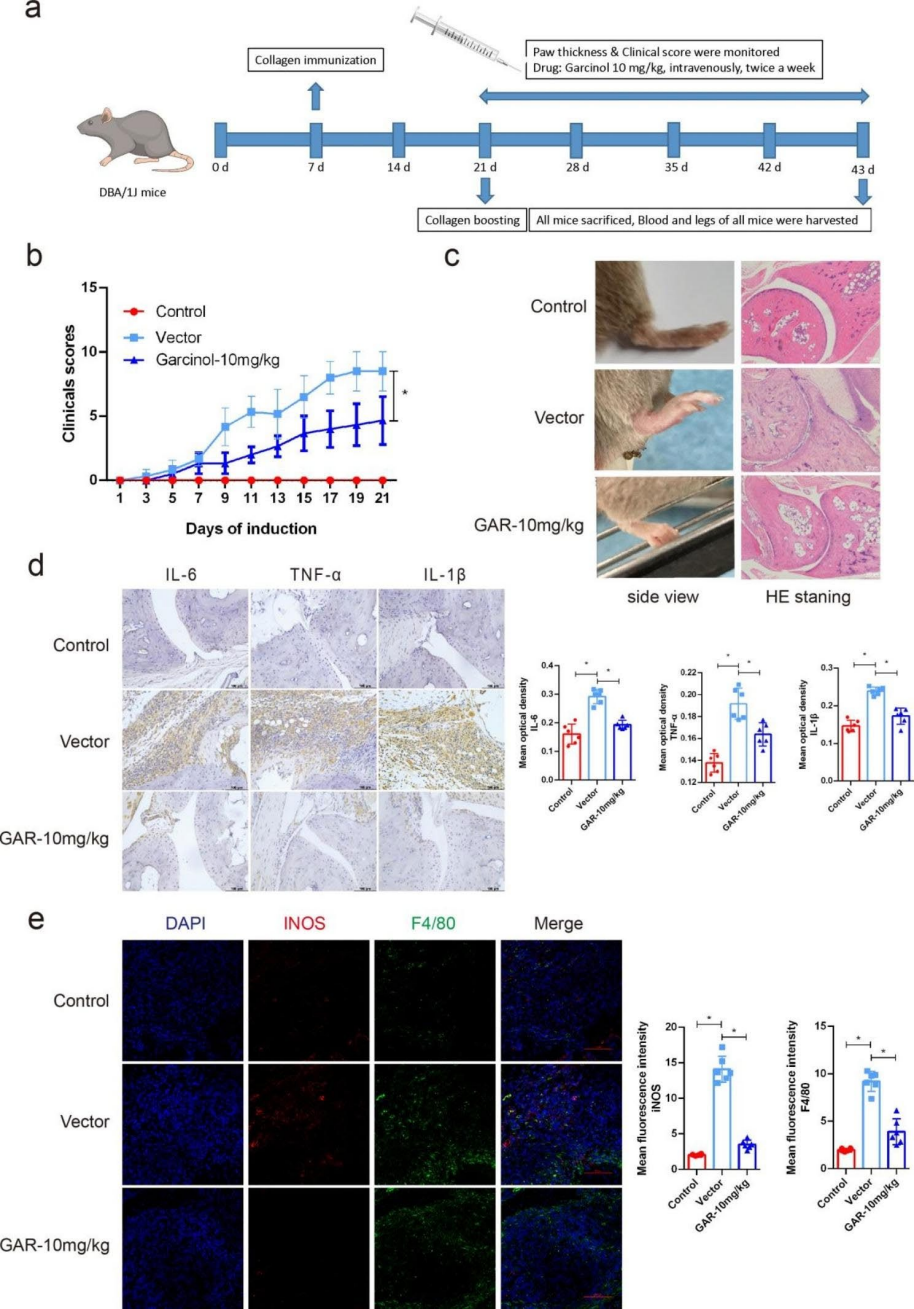
The natural compound garcinol, a PCAF inhibitor, significantly alleviated joint inflammation in CIA mice in vivo. (**a**) Schematic of CIA mice treated with garcinol. (**b**) Arthritis severity was evaluated using an arthritis scoring system in CIA mice treated with vector or 10 mg/kg garcinol (n = 6). (**c**) The degree of ankle swelling in CIA mice treated with vector or 10 mg/kg garcinol and H&E staining of the ankle joints excised from the mice are shown. (**d**) The expression of IL-6, TNF-α and IL-1β in CIA mice treated with vector or 10 mg/kg garcinol was detected by IHC. Semiquantitation of the mean optical density of IL-6, TNF-α and IL-1β determined by IHC analysis (n = 6). (**e**) The expression of iNOS in synovial macrophages of CIA mice treated with vector or 10 mg/kg garcinol were detect by IF (F4/80 green, iNOS red). Semiquantification of the mean fluorescence intensity of iNOS and F4/80 determined by IF (n = 6). ns indicates *P* > 0.05, and * indicates *P* < 0.05

To further study the therapeutic effect of targeting PCAF on autoimmune arthritis, we randomly divided 20 DBA mice aged 8–12 weeks into 2 groups (Fig. 3a). After boost immunization, they were given normal vector or 10 mg/kg garcinol intravenously. Arthritis scores were calculated every 2 days after administration. As shown by the results, 10 mg/kg garcinol treatment effectively reduced the arthritis scores of the CIA mice (Fig. 3b). In addition, joint swelling was significantly alleviated compared with that in the vector group (Fig. 3c). To further investigate the degree of inflammation, the mice were sacrificed, and paraffin sections were taken from the hind paws 23 days after boost immunization for HE and IHC staining. As expected, HE staining showed that the inflammatory infiltration of the joints was also significantly reduced after administration compared with that of the vector group (Fig. 3c). In addition, the levels of TNF-α, IL-6 and IL-1β in the treated group were significantly lower than those in the vector group, as shown by IHC staining (Fig. 3d). Semiquantification of the mean optical density values from IHC analysis also showed significant differences (Fig. 3d). Moreover, the IF results also demonstrated that M1 macrophage numbers at the arthritis site were significantly lower in the garcinol-treated mice than in the vector mice (Fig. 3e). These results indicated that PCAF plays a role in autoimmune arthritis and is a potential valuable therapeutic target.

### PCAF inhibition significantly interfered with the NF-κB pathway

**Fig. 4:**
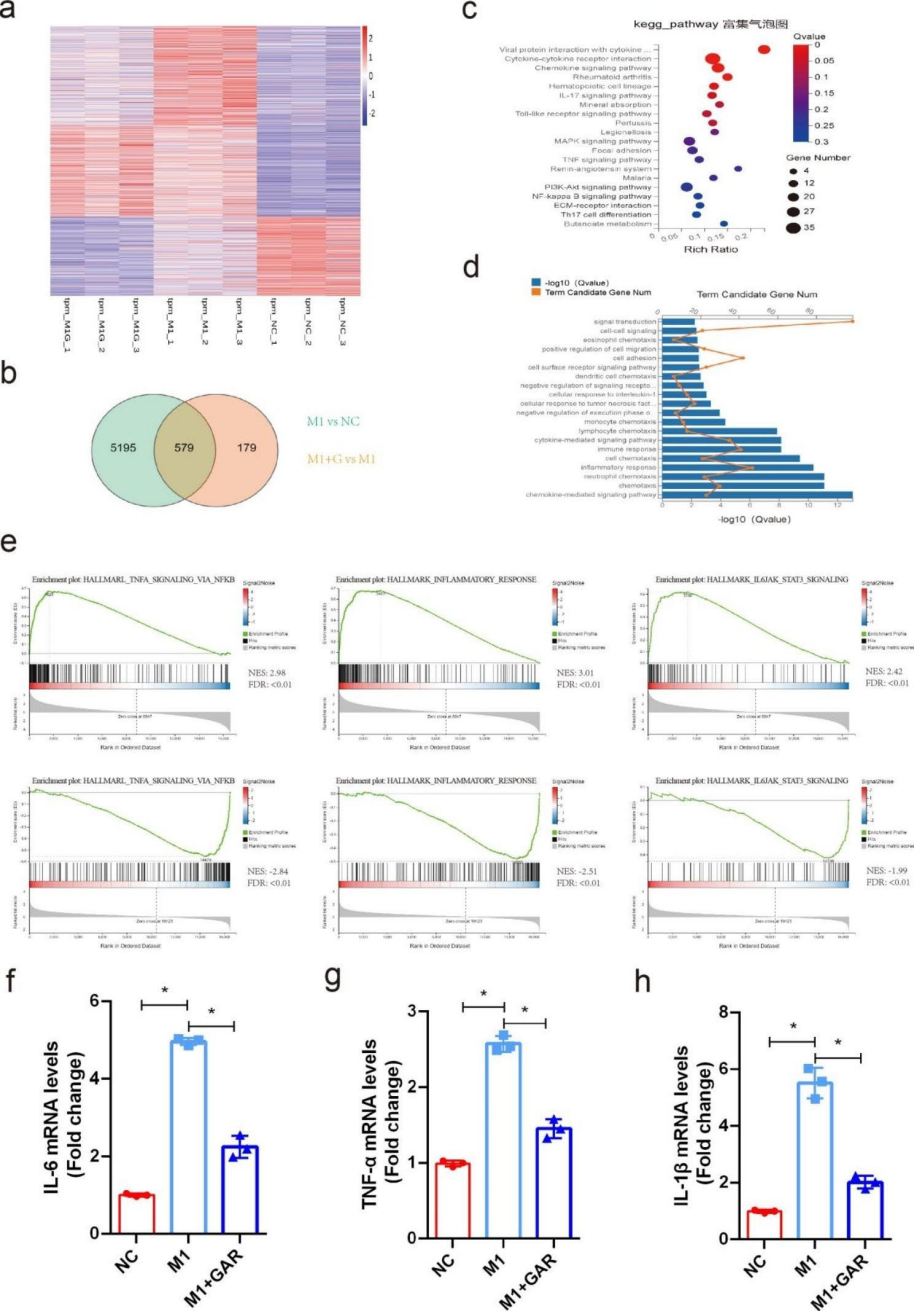
PCAF inhibition significantly interfered with the NF-κB pathway. (**a**) Cluster heatmap of differentially expressed genes in the control group, M1 group and garcinol group (n = 3 for each group). (**b**) Venn diagrams comparing the M1 group vs. the control group and the garcinol group vs. the M1 group. (**c**) Pathways that were significantly enriched in the 579 critical genes identified by KEGG analysis are shown. (**d**) Biological processes that were significantly enriched in the 579 critical genes identified by GO analysis are shown. (**e**) GSEA of genes related to TNF-α signaling via NF-κB, inflammation and the IL-6-JAK-STAT3 pathway. (**f**) The mRNA level of IL-6 in M1 macrophages was decreased after 20 µM garcinol treatment (n = 3). (**g**) The mRNA level of TNF-α in M1 macrophages was decreased after 20 µM garcinol treatment (n = 3). (h) The mRNA level of IL-1β in M1 macrophages was decreased after 20 µM garcinol treatment (n = 3). ns indicates *P* > 0.05, and * indicates *P* < 0.05

Whole-transcriptome sequencing of 3 repeats of M0, 3 repeats of M1 without 20 µM garcinol and 3 repeats of M1 with 20 µM garcinol was performed. When the M0 group and M1 group were compared, a total of 5774 differentially expressed mRNAs were found, while comparison of the M1 group and garcinol group revealed a total of 758 differentially expressed mRNAs (Fig. 4a). The mRNAs that were differentially expressed between the M0 and M1 groups and between the M1 and garcinol groups were considered critical genes. Through Venn diagram analysis, 579 critical differentially expressed mRNAs were identified (Fig. 4b). Moreover, “rheumatoid arthritis”, the “TNF signaling pathway” and the “NF-kappa B signaling pathway” were also enriched pathways according to KEGG analysis (Fig. 4c). As determined by GO analysis, these 579 mRNAs were enriched in the inflammatory response, chemotaxis and the chemokine-mediated signaling pathway in the biological process category (Fig. 4d). Moreover, gene set enrichment analysis (GSEA) showed that hallmarks, including TNF-α signaling via NF-κB, the inflammatory response and IL-6-JAK-STAT3 signaling related to the inflammatory pathway, were significantly enriched and upregulated in the M1 group compared with the M0 group but reduced in the M1 group compared with the garcinol groups (Fig. 4e). Consistent with the sequencing data, the qRT‒PCR results confirmed that the mRNA levels of the inflammatory factors TNF-α, IL-6 and IL-1β increased significantly during M1 polarization but rapidly decreased after PCAF inhibition (Fig. 4f-h). NF-κB plays an essential role in M1 polarization and subsequent inflammatory cytokine release. Our above results indicated that PCAF inhibition suppressed M1 polarization via strong NF-κB pathway interference. Thus, these results further support the role of PCAF in regulating M1 polarization.

### PCAF inhibition synergistically suppressed the acetylation of p65 and H3K9

**Fig. 5:**
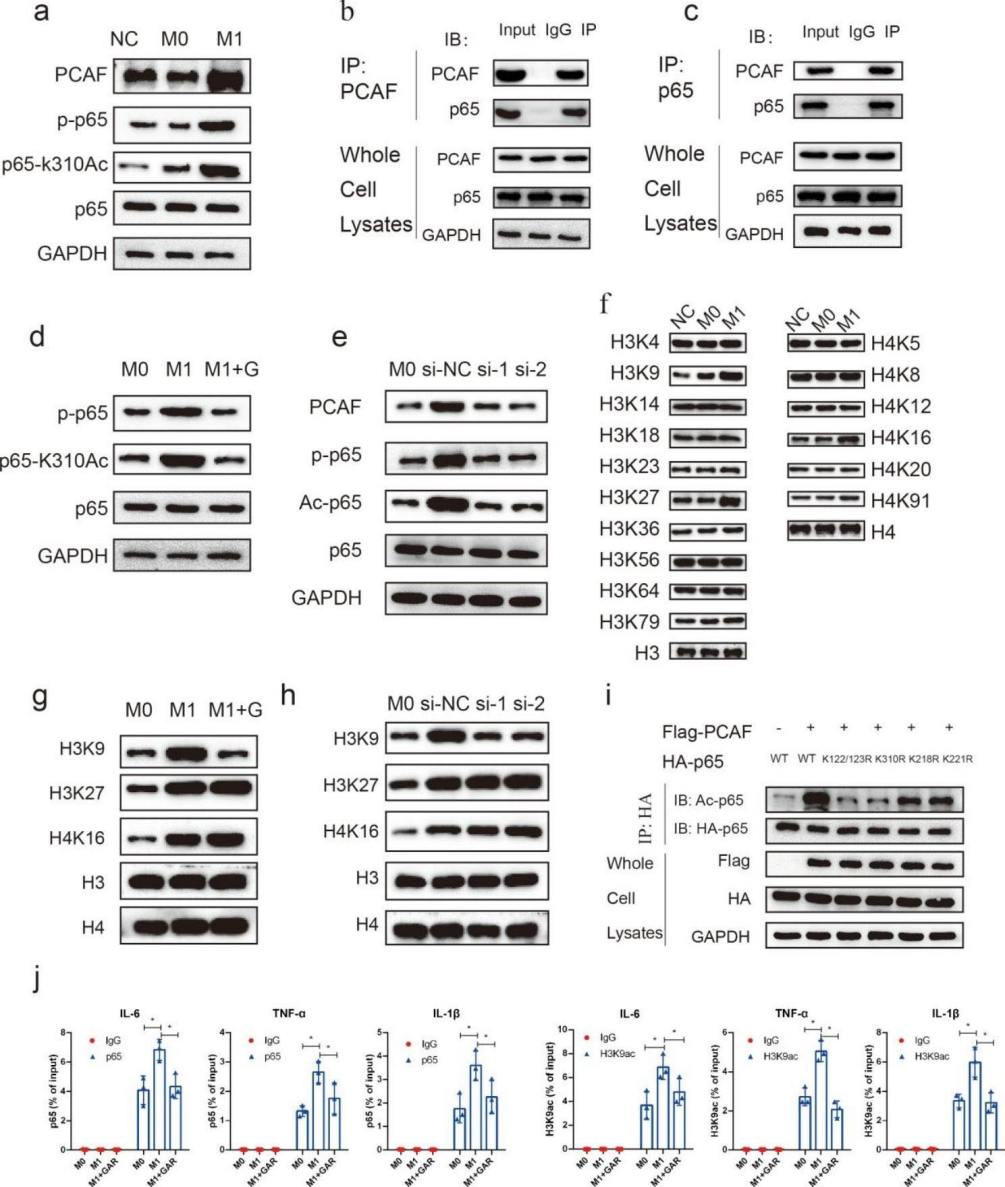
PCAF inhibition synergistically suppressed the acetylation of p65 and H3K9. (**a**) The protein levels of PCAF, p-p65, p65-K310Ac and p65 during the M1 polarization of macrophages were detected by western blotting (n = 3). (**b** & **c**) The binding of p65 and PCAF was assessed by co-IP assay (n = 3). (**d**) The levels of p-p65 and Ac-p65 (K310) were reduced by garcinol treatment (n = 3). (**e**) The levels of PCAF, p-p65, and Ac-p65 (K310) were reduced by PCAF siRNA knockdown (n = 3). (**f**) Changes in the abundance of various types of histone acetylation during the M1 polarization of macrophages were detected by western blotting (n = 3). (**g**) Changes in H3K9Ac, H3K27Ac and H4K16Ac levels after garcinol treatment are shown (n = 3). (**h**) Changes in H3K9Ac, H3K27Ac and H4K16Ac levels after PCAF siRNA knockdown are shown (n = 3). Changes in the acetylation level of p65 before and after the mutation of different residues (n = 3). (**i**) Changes in the acetylation level of p65 after PCAF overexpression and p65 mutations. (**j**) Changes in the binding of p65 and H3K9Ac at the promoters of TNF-α, IL-6 and IL-1β after garcinol treatment (n = 3). ns indicates *P* > 0.05, and * indicates *P* < 0.05

To further explore the role of PCAF in NF-κB activation during M1 polarization, we observed the level of p65 activation upon garcinol treatment during M1 polarization. Interestingly, the protein level of PCAF was found to be significantly upregulated during M1 polarization, and the phosphorylation and acetylation of K310 of p65 were also significantly enhanced (Fig. 5a). Previous studies have confirmed that PCAF can interact with p65. To further verify their interaction, reciprocal Co-IP assays were conducted. Co-IP and western blot assays confirmed the binding between PCAF and p65 (Fig. 5b-c). Furthermore, the phosphorylation level of p65 and the acetylation level of K310 were downregulated after garcinol treatment (Fig. 5d). To further verify the inhibitory effect of garcinol, siRNA was used to knock down PCAF, and similar results were obtained (Fig. 5e). K310 acetylation of p65 is indispensable for p65 activation; thus, these results demonstrated that PCAF regulates p65 activation through its pivotal role in p65 acetylation.

PCAF is an acetyltransferase that plays an important role in the acetylation of both nonhistone and histone proteins. Histone acetylation is necessary during gene transcription activation. Therefore, we further investigated the alteration of histone acetylation during the polarization of M1 macrophages. The acetylation of H3K9, H3K27 and H4K16 was remarkably augmented during M1 polarization, while other types of histone acetylation were not significantly altered (Fig. 5f). Furthermore, we found that garcinol treatment significantly diminished the acetylation level of H3K9 but did not affect the acetylation level of H3K27 or H4K16 (Fig. 5g). Similar results were also observed in the siRNA knockdown assay (Fig. 5h). Previous studies have reported that p65 can be acetylated not only at K310 but also at other residues, including K218, K221 and K122/123. To further study the influence of PCAF on the acetylation of p65, we constructed specific lysine-alanine mutants of p65 at K310, K218, K221, and K122/123 and conducted IP assays. The results showed that the acetylation level of p65 was remarkably augmented after the overexpression of PCAF, while the acetylation level was diminished after p65 mutation at all these sites, suggesting that PCAF acetylates p65 at all these residues (Fig. 5i). Finally, we conducted ChIP‒qPCR and found that p65–H3K9Ac binding was elevated at the promotors of TNF-α, IL-6 and IL-1β during M1 polarization, while garcinol treatment significantly decreased the binding signals (Fig. 5j).

Collectively, these results indicated that PCAF inhibition suppressed M1 polarization and subsequent inflammatory cytokine release at least partially through synergetic NF-κB and H3K9Ac blockade.

### DSNPs can be selectively absorbed by activated macrophages

**Fig. 6:**
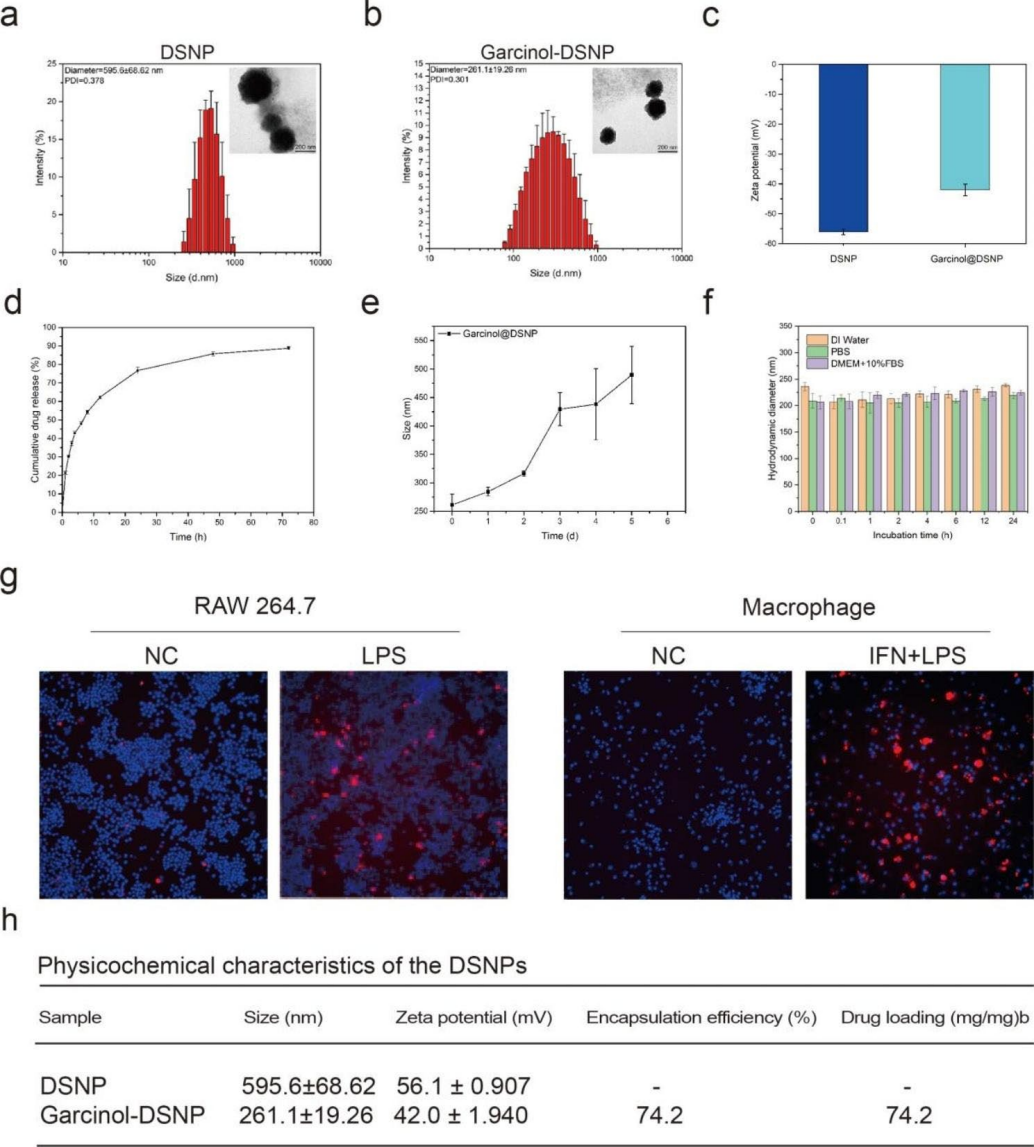
DSNPs could be selectively absorbed by activated macrophages. (**a**, **b**) Size distributions of DSNPs and garcinol-DSNPs in distilled water at 25 ℃ and TEM images are shown. (**c**) Zeta potential values of the DSNPs and garcinol-DSNPs. (**d**) Profile showing in vitro release of garcinol from DSNPs in PBS. (**e**) Curve showing the change in garcinol-DSNP size over 5 days. (**f**) The stability of garcinol-DSNPs in serum was determined by DLS. (**g**) In vitro cellular uptake of DSNPs by RAW264.7 cells (LPS±) and human-derived macrophages (LPS±/IFN-γ±). (**h**) Summary of the physicochemical characteristics of garcinol-DSNPs and DSNPs

DS and its derivatives exhibit remarkable biocompatibility and biodegradability. Furthermore, DS exhibits ideal activated macrophage targeting via receptor‒ligand interactions. Thus, we designed DSNPs carrying garcinol for PCAF inhibition in vivo. As verified by infrared spectroscopy and nuclear magnetic resonance, DSNPs were successfully synthesized (Supplementary Fig. 3a, b). A map showing the distribution of DSNP and garcinol-DSNP particle size in water indicated that the DSNPS particle size was 595.6 ± 68.62 nm (Fig. 6a). After garcinol loading, the particle size of garcinol-DSNPs decreased to 261.1 ± 19.26 nm (Fig. 6b), suggesting that dense nanoparticles formed after garcinol loading. The zeta potential values of the DSNPs and garcinol-DSNPs were − 56.1 ± 0.91 mV and − 42 ± 1.94, respectively (Fig. 6c). Next, the in vitro drug-release kinetics of the nanoparticles were determined over 3 days in PBS (pH 7.4). Garcinol was rapidly released within the first 6 h (47.89 ± 0.95%) and then continued to be released for another 3 days. At the end of the tests, the cumulative release was 85.72 ± 1.10% (Fig. 6d). Furthermore, the change in size over 5 days was also assessed, and the results indicated that over time, the particle size of the garcinol-DSNPs gradually increased, which might be due to gradual release of garcinol from the nanoparticles. When garcinol was thoroughly released, the particle size of the nanoparticles returned to that of the DSNPs (Fig. 6e). Moreover, the garcinol-loading capacity of the prepared garcinol-DSNPs was 74.2 µg/mg, and the encapsulation rate was 74.2% (Supplementary Fig. 3c). To characterize the stability of garcinol-DSNPs in serum, dynamic light scattering (DLS) was conducted. Basic DMEM culture medium with 10% FBS was used to imitate the blood environment in vivo. As the results showed, garcinol-DSNPs maintained a stable size in DI water, PBS and DMEM with 10% FBS for 24 h, which confirmed the excellent stability of garcinol-DSNPs in serum (Fig. 6f).

To further evaluate the cellular uptake of the DSNPs, RAW264.7 macrophages and human-derived macrophages were treated with cy5.5-labeled DSNPs (red). After the RAW264.7 cells (LPS±) and human-derived macrophage cells (LPS±/IFN-γ±) were incubated with DSNPSs at 37 ℃ for 1 h, strong fluorescence signals were detected in the RAW264.7 cells (LPS+) and macrophages (LPS+/IFN-γ+), and significantly weaker fluorescence signals were observed in RAW264.7 cells (LPS-) and macrophages (LPS-/IFN-γ-), which were used as controls (Fig. 6g). The physicochemical characteristics of garcinol-DSNPs are summarized in Fig. 6h.

In summary, the prepared garcinol-DSNPS showed good chemical and physical properties and was effectively internalized by activated macrophages.

### Garcinol-DSNPs ideally target inflammatory joints and effectively alleviate arthritis

**Fig. 7:**
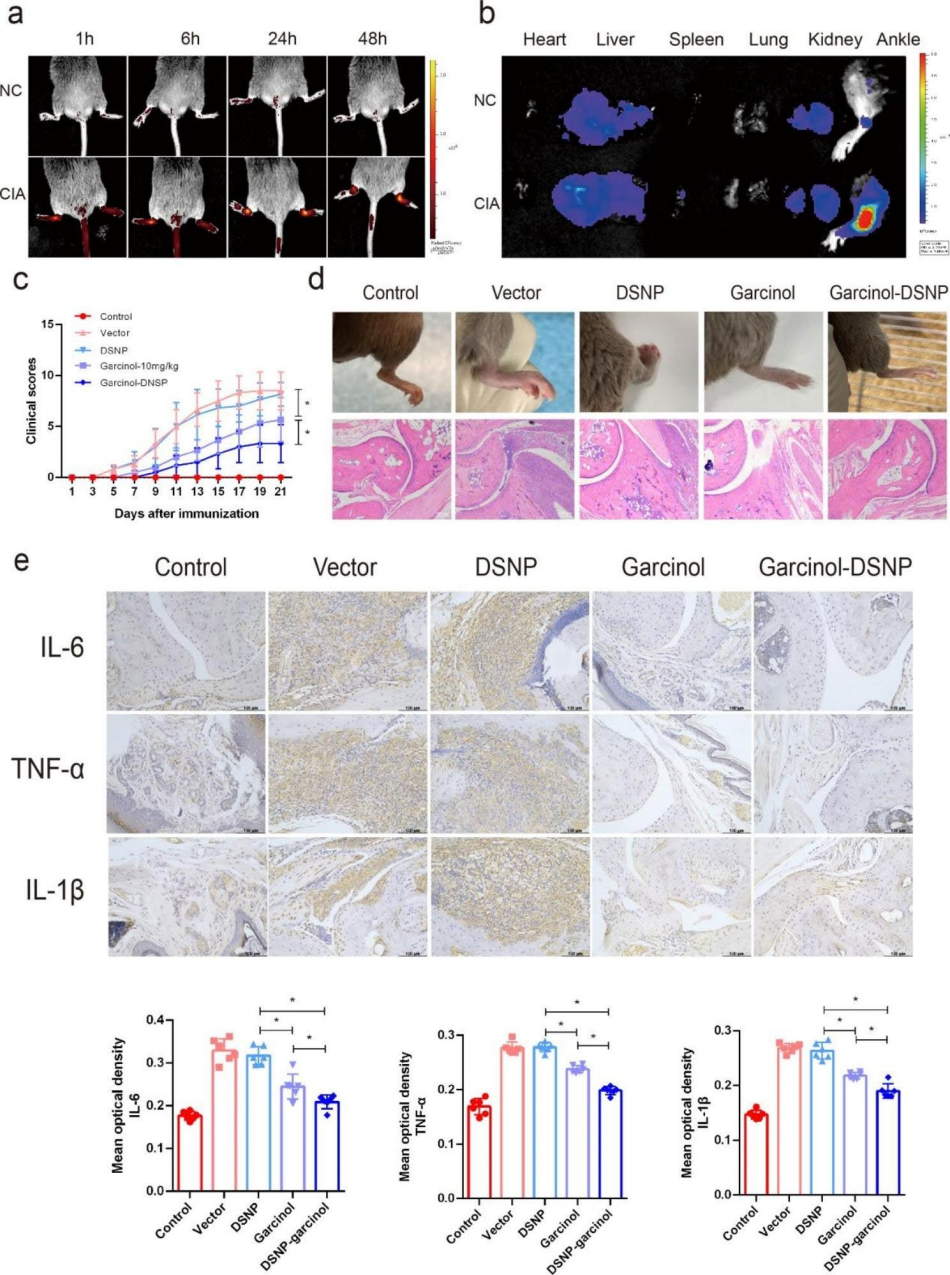
Garcinol-DSNPs ideally targeted inflammatory joints and effectively alleviated arthritis. (**a**) The time-dependent distribution of DSNPs in images of the whole hind legs of control and CIA mice was evaluated with an IVIS system. (**b**) Ex vivo distribution of DSNPs in various organs from control and CIA mice. (**c**) Arthritis severity was evaluated using an arthritis scoring system in CIA mice treated with vector, DSNPS, 10 mg/kg garcinol or 10 mg/kg garcinol-DSNPS (n = 6). (**d**) The general degree of ankle swelling in CIA mice treated with vector, DSNPs, 10 mg/kg garcinol or 10 mg/kg garcinol-DSNPs and H&E staining of ankle joints excised from the different groups of mice are shown. (**e**) The expression of IL-6, TNF-α and IL-1β in CIA mice treated with vector, DSNP, 10 mg/kg garcinol or 10 mg/kg garcinol-DSNP was detected by IHC. Semiquantification of the mean optical density of IL-6, TNF-α and IL-1β detected by IHC (n = 6). ns indicates *P* > 0.05, and * indicates *P* < 0.05

To further assess whether DSNPs loaded with garcinol could reach active inflammatory sites, the biodistribution of cy5.5-labeled DSNPs was observed in vivo in wild-type and CIA model mice by an IVIS in vivo imaging system. The fluorescence intensity of the legs of wild-type mice did not change significantly over 48 h of observation, while in the CIA model mice, fluorescence was detected after 1 h, peaked at 6 h, and then gradually decreased (Fig. 7a). In addition, we detected the fluorescence intensity of ex vivo organs from the WT and CIA mice 48 h later, and the results indicated obvious fluorescence signals in the livers and kidneys of the WT and CIA mice, but the intensities were not significantly different between the groups. Interestingly, a strong fluorescence signal was detected in the legs of the CIA mice, but this was not evident in the legs of the WT mice (Fig. 7b). These results suggested that DSNPs targeted the inflammatory joint sites in the CIA mice well.

To further evaluate the anti-inflammatory effect of DSNPs loaded with garcinol, a CIA mouse model was constructed. Garcinol-DSNPs (10 mg/kg garcinol) were administered twice a week into the tail vein of each mouse, and vector, unloaded DSNPs, and free garcinol (10 mg/kg garcinol) were used as controls. Compared with those of the vector and unloaded DSNP groups, the arthritis symptoms and joint score were significantly decreased in the garcinol group, as expected (Fig. 7c, d). Moreover, garcinol-DSNP treatment in the mice had a better effect than even garcinol alone (Fig. 7c, d). HE staining of the major organs of the mice showed that DSNPs, garcinol alone or DSNPs loaded with garcinol caused no significant damage to the major organs of the mice (Supplementary Fig. 4).

In addition, we further evaluated the expression of classic inflammatory factors in the inflammatory ankles of the mice in each group. The results demonstrated that garcinol-DSNP treatment more effectively inhibited TNF-α, IL-6 and IL-1β expression than garcinol alone (Fig. 7e). Semiquantification of the mean optical density values from IHC analysis also showed significant differences (Fig. 7e). Furthermore, immunofluorescence assay was conducted to assess NF-kB activation, H3K9 acetylation and M1 polarization in synovial macrophages. Compared with those in the vector group and the unloaded DSNP group, the phosphorylation and acetylation of K310 of p65, the acetylation of H3K9 and the M1 polarization in macrophages were downregulated in the free garcinol group, which indicated that targeting macrophage M1 polarization suppression through PCAF inhibition alleviates autoimmune arthritis at least partially via synergistic NF-κB and H3K9Ac blockade. Moreover, garcinol-DSNP treatment showed more effective inhibition, suggesting that DSNPs loaded with garcinol alleviated the arthritis more effectively (Supplementary Fig. 5).

In summary, these results indicated that DSNPs loaded with garcinol could effectively target the inflammatory joint site and had a significant therapeutic effect on arthritis.

Autoimmune rheumatic diseases, including RA, ankylosing spondylitis, systemic lupus erythematosus, and systemic sclerosis, are a set of diseases characterized by an abnormal immune system response, cytokine dysregulation and the presence of a continuous proinflammatory microenvironment in vivo [37, 38].

More than 5% of the global population suffers from autoimmune rheumatic diseases [38]. Although these disorders can affect different organs and although their clinical presentations can be heterogeneous, their pathogenic mechanism and pathologic features exhibit similarities [37, 38].

Several seminal clinical observations provided the first evidence of the key contribution of macrophages to the pathogenesis of autoimmune rheumatic diseases [39]. Macrophages are generally divided into proinflammatory M1-type and anti-inflammatory M2-type macrophages [8, 9]. M1/M2 polarization imbalance is a pathological feature of most autoimmune rheumatic diseases [8]. M1 macrophages not only directly secrete several proinflammatory factors, such as TNF-α, IL-1β and IL-6, to aggravate the proinflammatory microenvironment but also indirectly facilitate the differentiation of other adaptive immune cells, such as Th1 and Th17 cells, for inflammation induction or maintenance [2, 8, 9]. Thus, reversing the M1/M2 imbalance is a rational and promising strategy for treating autoimmune rheumatic diseases. Joints are commonly susceptible to several autoimmune rheumatic diseases. Sustained inflammatory invasion leads to joint damage and progressive disability [6, 7]. RA is the most common type of autoimmune arthritis worldwide [40]. More than 1% of the global population suffers from RA [41]. Moreover, mature animal models of RA have been developed over the past decades. The CIA mouse model is the most common classic animal model used to study RA [32, 33]. M1 macrophage polarization is pivotal for the initiation and maintenance of arthritis in RA patients and CIA mice [8, 32]. Thus, in this study, we collected RA patient specimens and constructed CIA mice for further study of M1 polarization.

PCAF inhibition has potent anti-inflammatory effects [14, 42, 43]. However, whether PCAF can act as an effective target for inhibiting M1 macrophage polarization and treating autoimmune arthritis has not been systematically studied in vivo. In our study, we demonstrated that PCAF, rather than its intimate “collaborator” GCN5, p300 or CBP [44], was overexpressed in activated M1 macrophages both in vitro and in vivo, which indicated a specific and important role for PCAF in M1 polarization. We further demonstrated that PCAF knockdown or inhibition could effectively inhibit M1 macrophage polarization and proinflammatory cytokine release both in vitro and in vivo. Arthritis severity in the inflamed joints was accordingly alleviated after PCAF knockdown or inhibition. Thus, PCAF can be regarded as a therapeutic target for the treatment of M1 macrophage-related autoimmune rheumatic diseases with great potential.

NF-κB activation plays a pivotal role in both M1 macrophage polarization and downstream proinflammatory cytokine release [45, 46]. In this study, we demonstrated that PCAF knockdown or inhibition impaired NF-κB activation via p65 deacetylation. Acetylation plays a dual role in NF-κB activation [46]. Acetylation of K221 increases the binding affinity of p65 for the κB enhancer and, together with acetylation of K218, prevents the association of p65 with IκBα to promote the transcriptional activity of NF-κB [46]. In contrast, acetylation of K310 in p65 does not control DNA binding or IκBα assembly but is required for the full transactivation function of NF-κB [46]. Conversely, acetylation of K122/123 in p65 may impair overall transcriptional activity [15]. In this study, we demonstrated that knockdown or inhibition of PCAF deacetylated p65 at all of the abovementioned lysine residues. These results indicated that PCAF induced panacetylation of p65. The overall effect of PCAF was to promote the activation of NF-κB, although the acetylation of some lysine residues by PCAF might counter this function. The regulatory relationship between PCAF and NF-κB may warrant further exploration. Furthermore, we demonstrated that PCAF knockdown or inhibition also reduced the H3K9Ac level in macrophages. H3K9Ac is generally linked to transcriptional gene activation [47, 48]. ChIP‒qPCR data further demonstrated that PCAF inhibition led to a reduction in NF-κB transcriptional activity coupled with a decrease in H3K9Ac levels at several genes that modulate M1 polarization and encode inflammatory cytokines. Thus, our study indicated that in addition to direct inhibition of the acetylation and activation of p65, PCAF knockdown or inhibition reduced stimulus-coupled H3K9Ac modification surrounding NF-κB target genes to control overall NF-κB-mediated M1 macrophage polarization and inflammatory cytokine transcription. This anti-inflammatory effect of PCAF inhibition is primarily mediated through NF-κB activity modulation. A strategy that combines PCAF inhibition with TNF-α or IL-17 A neutralization may result in a more efficient clinical outcome in the treatment of autoimmune rheumatic diseases.

In this study, we chose to inhibit PCAF with garcinol in vivo. Garcinol is a natural polyisoprenylated benzophenone derivative from the *Garcinia indica* fruit rind [49]. Plant extracts containing garcinol have been used for centuries as Chinese traditional medicine and are considered safe and effective in treating inflammatory diseases [50]. Thus, the pure form of garcinol shows great potential in the treatment of inflammatory diseases as an epigenetic therapeutic agent in the clinic [50–52]. In this study, we first validated that garcinol could effectively inhibit M1 macrophage polarization in vivo and significantly alleviate inflammation severity in the joints of CIA mice. However, similar to most other drugs used in the clinic to treat autoimmune rheumatic diseases, garcinol showed an extensive and nonspecific distribution in vivo, which may cause serious side effects that impede its application in the clinic.

Targeting activated macrophages in the inflammatory environment via receptor‒ligand interactions has been proven to be effective and applicable in the clinic over the past few years [27, 53]. SR-A is specifically overexpressed in activated macrophages [29, 54, 55], in which SR-A interacts with its ligand to mediate endocytosis [55]. Based on these findings, nanoparticles containing packaged SR-A ligands could deliver drugs into activated macrophages via endocytosis activation [29, 56]. The targeting of SR-A on activated macrophages has been proven to be even more superior than that of the class CD44 receptor for drug delivery [56]. DS and its derivatives show remarkable biocompatibility and biodegradability [29]. Additionally, DS exhibited ideal activated macrophage targeting via receptor‒ligand interactions with SR-A [29]. Thus, we designed DSNPs carrying garcinol to inhibit PCAF in vivo. This strategy achieved effective targeted delivery of garcinol to activated M1 macrophages, which facilitated the delivery of garcinol to M1 macrophage-enriched inflamed regions and subsequently led to excellent therapeutic outcomes with few side effects.

## Conclusions

Together, the results of our proof-of-principle study indicate that targeted PCAF inhibition with the natural compound garcinol in DSNPs might be a promising strategy for the treatment of autoimmune arthritis via ideal targeted M1 macrophage polarization inhibition through synergistic NF-κB and H3K9Ac blockade.

## Materials and methods

### Ethical statement

The mouse experiments were approved by the Institutional Animal Care and Use Committee of Sun Yat-Sen University, Guangzhou, China (ethical approval numbers: 2,021,002,119, 2,022,000,246). All experimental procedures in the mice strictly adhered to rules and guidelines for the ethical use of animals in research.

### Patients

The Eighth Affiliated Hospital of Sun Yat-Sen University approved this study (ethical approval number: 2020r132). After informed consent was obtained, synovial tissues were obtained from 6 osteoarthritis (OA) and 6 rheumatoid arthritis (RA) patients undergoing artificial arthroplasty at the Eighth Affiliated Hospital of Sun Yat-Sen University. Rheumatoid factors (RFs), erythrocyte sedimentation rate (ESR), C-reactive protein (CRP) and antibodies against cyclic citrullinated peptide (anti-CCP Ab) were examined. The characteristics of the OA and RA patients are listed in Table 1.

**Table 1:** Characteristics of the OA and RA patients

	OA (n = 6)	RA (n = 6)
Age, years (± SD)	62.2 (± 4.1)	59.7 (± 5.5)
Sex, female	4	4
Disease duration, years (± SD)	-	15.7 (± 4.2)
RF, IU/mL (± SD)	-	161.2 (± 57.1)
CRP, mg/mL (± SD)	3.2 (± 4.5)	14.6 (± 11.4)
ESR, mm/h (± SD)	27.4 (± 6.3)	33.9 (± 8.1)
Anti-CCP ab, U/mL (± SD)	-	402.7 (± 136.4)

### Animals

All animal experimental procedures were approved by the Institutional Animal Care and Use Committee of Sun Yat-sen University.

PCAF-/- mice (12–14 weeks old) were generated by Cyagen Bio, Guangzhou, and all mice were maintained in a specific pathogen-free facility. CIA was induced through booster immunization with high-dose adjuvant. Briefly, 100 µL of an emulsion containing chicken collagen II (C II, Chondrex 20,012, 2 mg/ml) and Freund’s complete adjuvant (CFA, Chondrex 7023, 5 mg/ml) was injected intradermally to the end of the tail. A booster injection was repeated on day 21.

DBA mice (8–12 weeks old) were generated by Gem Pharmatech by Jiang Su, and all mice were maintained in a specific pathogen-free facility. For CIA induction, 100 µL of an emulsion containing C II (Chondrex, 20,012, 2 mg/ml) and CFA (Chondrex, 7001, 4 mg/ml) was injected intradermally to the end of the tail. For booster injection, 100 µL of an emulsion containing C II and incomplete Freund’s adjuvant (Chondrex IFA, 7002) was administered subcutaneously on day 21. Additionally, on day 21, these CIA mice were treated with 100 µL of vector (dimethyl sulfoxide, DMSO, 0.01% v/v), 100 µL of garcinol (10 mg/kg, dissolved in DMSO, 0.01% v/v, a conventional dose used in mice in vivo [34–36]), 100 µL of DSNPs (dissolved in PBS), and 100 µL of garcinol-DSNPs (10 mg/kg, dissolved in PBS) intravenously twice a week according to the grouping.

All animals were euthanized on day 43. Arthritis severity was assessed using an established semiquantitative scoring system of 0–4, where 0 = normal, 1 = mild swelling, 2 = moderate swelling, 3 = swelling of all joints and 4 = joint distortion and/or rigidity and dysfunction. The cumulative score for all four paws of each mouse (maximum possible score of 16) was used as the arthritis score to represent overall disease severity and progression in an animal.

### Preparation of amphiphilic DSNPs

First, 5β-cholic acid (1 g, 2.8 mmol) was dissolved in 5 ml of methanol, and concentrated hydrochloric acid (180 ml, 4.9 mmol) was added. The solution was stirred at reflux at 60 °C for 6 h. It was then cooled to 0 °C to crystallize to obtain a precipitate, which was filtered through a membrane filter (pore size of 0.45 mm, Micropore) and washed with cold methanol. The product was dried under vacuum at room temperature to give methyl 5b-cholate. It was then dissolved in ethylenediamine, refluxed at 130 °C for 6 h, and cooled to room temperature until precipitation occurred. The precipitate was filtered through a 0.45-mm membrane filter and washed thoroughly with water. The samples were vacuum dried at 40 °C for 24 h to obtain aminoethyl 5β-cholic acid. Then, dextran sulfate (DS, 100 mg, 0.262 mmol) and 4-nitrophenyl chloroformate (158 g, 0.786 mmol) were dissolved in DMSO/pyridine (15 mL, 1/1 (v/v)), and 4-dimethylamino-pyridine (9.6 mg, 0.0786 mmol) was used as the catalyst. The mixture was stirred at 0 °C for 4 h under nitrogen, and the product was precipitated in 300 ml of ether/ethanol (1/1, v/v), filtered and washed with sufficient ether. Then, the product was dried at 40 °C for 6 h to obtain 4-nitrophenyl-activated DS. Finally, the aminoethyl 5β-cholic acid (13.81 mg, 0.0356 mmol) prepared as described above was added to 10 mL of DMSO/pyridine (2/1, v/v), and the 4-nitrophenyl-activated DS prepared as described above was subsequently added (100 mg, 0.178 mmol). The mixture was stirred at 50 °C for 48 h under argon. After the solution was concentrated, the excess water was dialyzed using a dialysis membrane (molecular weight cutoff, MWCO = 12–14 kDa) for 72 h and lyophilized to obtain amphiphilic DSNPs. The source and purity of the reagents used are shown in Table S1.

### Preparation of garcinol-DSNPs

Amphiphilic DSNPs (100 mg) were dissolved in 10 ml of tetrahydrofuran/distilled water (1/1, v/v). After adding garcinol (10 mg), the mixture was sonicated at 4 °C for 30 min and dialyzed with distilled water (MWCO = 6–8 kDa) for 12 h to remove the free garcinol. The purified solution was filtered (0.8-mm syringe) and lyophilized to yield a pale-yellow powder consisting of garcinol-DSNPs.

### Isolation and culture of PBMCs

Peripheral blood mononuclear cells (PBMCs) were derived from peripheral blood donated by 18 healthy volunteers. The intermediate buffy coat was obtained by density gradient centrifugation, and the buffy coat was extracted into a centrifuge tube with PBS and washed twice, after which the PBMCs were counted and resuspended in serum-free 1640 medium (Gibco, C11875500BT). Subsequently, 10^6^ cells per well were seeded in a 12-well plate and incubated at 37 °C for 4 h. Then, the supernatant was discarded, and the wells were washed carefully with PBS. Then, 10% FBS-containing 1640 medium supplemented with 25 ng/ml M-CSF (PeproTech, 300-25-10) was added to the cells, which were cultured at 37 °C. The medium was replaced every 3 days. To induce the formation of M1 macrophages, 1 mg/ml LPS (Sigma, L2880) and 30 ng/ml IFN-γ (Peprotech, 300-02-20) were added on the 6th day, and the cells were collected after culture for another 2 days for subsequent experiments. To induce the formation of M2 macrophages, 20 ng/ml IL-4 (Peprotech, 200-04-5) and 20 ng/ml IL-13 (Peprotech, 200-13-2) were added to the medium on the 6th day. The cells were collected for detection after culture for another 2 days.

### Culture of 293T cells

293T human embryonic kidney cells were cultured in glucose DMEM (Cienry, CR-12,800) with 10% FBS at 37 °C under 5% CO_2_. When the cells reached 80–90% confluence, the 293T cells were digested with 0.25% trypsin containing 0.53 mM EDTA and reseeded in new flasks.

### Tissue immunohistochemistry (IHC)

The obtained tissue samples were successively fixed, decalcified, and embedded in paraffin. The sections were deparaffinized and hydrated. For mouse tissue IHC assays, the sections were incubated in 10 mM citrate buffer and microwaved at 750 W for 30 min for antigen retrieval. The sections were treated with 3% H_2_O_2_ for 20 min and blocked with 5% normal goat serum for 1 h. Then, the sections were separately incubated with anti-PCAF (Santa Cruz, sc-13,124), anti-p300 (Abcam, ab275379), anti-CBP (Abcam, ab253202), anti-GCN5 (Santa Cruz, sc-365,321), anti-IL-6 (Abcam, ab-290,735), anti-IL-1β (Abcam, ab-283,818), or anti-TNF-α (Abcam, ab-1793) antibodies at 4 °C overnight. Secondary antibody incubation and color development were performed using the SP Rabbit & Mouse HRP DAB Kit (Cwbio, 2069 S) according to the kit protocol.

Immunohistochemistry (IHC) semiquantitative analysis was performed with ImageJ software. Briefly, color separation was first performed with the IHC Toolbox plugin and the H-DAB model. The image was then converted to 8-bit format. Next, image correction was performed with the “uncalibrated” function. The average optical density value was determined, and the mean optical density (Mean) was calculated with the following equation: Mean = integrated optical density (IntDen)/area of the entire image (Area).

### Tissue IF

The obtained tissue samples were successively fixed, decalcified, and embedded in paraffin. The sections were deparaffinized, hydrated, and incubated in 1% Triton X-100/PBS. After antigen retrieval in citrate buffer and blocking in goat serum, the sections were incubated with anti-CD68 (Abcam, ab213363), anti-PCAF (Santa Cruz, sc-13,124), anti-F4/80 (Abcam, ab300421), and anti-INOS (R&D, MAB9502) antibodies overnight at 4 °C. Then, the slides were stained with fluorophore-labeled secondary antibodies (1:500) for 1 h and DAPI for another 10 min to stain the cell nuclei. All images were obtained by a laser scanning confocal microscope (Nikon C2).

IF semiquantitative analysis was performed with ImageJ software: First, a single channel was extracted with the Image Color Split Channels function. The threshold was adjusted and the appropriate area selected with Image Adjust Threshold. Next, the appropriate threshold algorithm was selected from among all the available thresholds with Image Adjust Auto Threshold. The average fluorescence intensity was measured from the mean gray value with the threshold as the limit. Then, the mean fluorescence intensity (Mean) was calculated as follows: Mean = total fluorescence intensity of the selected area (IntDen)/area of the selected area (Area).

### Flow cytometry

To detect M1/M2 macrophages, the collected macrophages were incubated for 30 min at room temperature with the following specific antibodies: anti-human HLA-DR-PE (BD, 555,812) and anti-human CD206-APC (BD, 550,889). After three washes, the cells were incubated with a fixation/permeabilization working solution (eBioscience, 00-5523-00) in the dark for 60 min at room temperature. After another three washes, the cells were incubated with an anti-human CD68-FITC antibody (BD, 562,117) in the dark for 30 min. Then, the cells were resuspended in 200 µl of PBS and collected into tubes. All samples were analyzed using a BD FACSCelesta flow cytometer.

### Immunoprecipitation assays

The cells were quickly harvested and homogenized on ice in lysis buffer for Western and IP (Beyotime, P0013) plus 1 mM PMSF (Beyotime, ST506) and 1% phosphatase inhibitor (Boster, AR1183). After protein A + G magnetic beads were washed, antibodies against PCAF (Abcam, ab176316), p65 (Abcam, ab218533), HA (Cell Signaling Technology, 3724 S) and Flag (Cell Signaling Technology, 14,793 S) were incubated with the beads. Then, IgG from the corresponding species was used to eliminate nonspecific binding. Thereafter, protein A + G magnetic beads conjugated with antibody or normal IgG were added to the cell extracts and incubated according to the manufacturer’s instructions. Then, the magnetic beads were collected, washed, and resuspended in 60 µl of SDS loading buffer. All samples were boiled for 5 min and analyzed via western blotting.

### Quantitative real-time PCR (qRT‒PCR)

qRT‒PCR was performed as previously described. Briefly, total cellular RNA was isolated using TRIzol (Invitrogen, 15,596,018) and reverse transcribed into cDNA using PrimeScript™ reagent kits (TaKaRa, RR036A). qRT‒PCR was performed by using TB Green Premix Ex Taq (Takara, RR820A) according to the manufacturer’s instructions on a 7500 real-time PCR detection system (Applied Biosystems, Carlsbad, CA). Data were normalized to GAPDH data from control samples, and relative expression levels were analyzed using the 2^−∆∆^ Ct method. The primers specific for each gene are listed in Table S2.

### Western blotting

Cultured cells were washed with cold PBS and lysed in RIPA buffer (Cwbio, CW2333S) plus 1% PMSF and 1% phosphatase inhibitor. The lysate was centrifuged at 4 °C for 30 min, and the protein concentration in the supernatant was measured with a BCA assay kit (Cwbio, CW0014S). Equal amounts of each sample were diluted in 5 × SDS loading buffer (Beyotime, P0015), separated by 10% SDS–polyacrylamide gel electrophoresis (Beyotime, P0012) and then transferred to a polyvinylidene fluoride membrane (Millipore, IPVH00010). The membranes were blocked with 5% nonfat milk in TBST (150 mM NaCl, 0.05% Tween-20, and 50 mM Tris–HCl, pH 7.5) for 1 h at room temperature and incubated overnight at 4 °C with antibodies against GAPDH (Cell Signaling Technology, 5174), PCAF (Abcam, ab176316), p65 (Cell Signaling Technology, 8242 S), p-p65 (Cell Signaling Technology, 3033 S), p65-K310Ac (Abcam, ab218533), H3K4Ac (Abcam, ab176799), H3K9Ac (Abcam, ab32129), H3K14Ac (Abcam, ab52946), H3K18Ac (Abcam, ab40888), H3K23Ac (Abcam, ab177275), H3K27Ac (Abcam, ab177178), H3K36Ac (Abcam, ab177179), H3K56Ac (Abcam, ab238307), H3K64Ac (Abcam, ab214808), H3K79Ac (Abcam, ab214731), H3 (Abcam, ab201456), H4K5Ac (Abcam, ab51997), H4K8Ac (Abcam, ab45166), H4K12Ac (Abcam, ab177793), H4K16Ac (Abcam, ab109463), H4K20Ac (Abcam, 177,191), H4K91Ac (Abcam, ab4627), H4 (Abcam, ab177840), HA (Cell Signaling Technology, 3724 S) and Flag (Cell Signaling Technology, 14,793 S). The membranes were incubated with horseradish peroxidase (HRP)-conjugated anti-rabbit IgG (Boster, BA1054) or anti-mouse IgG (Boster, BA1050) diluted 1:3000 at room temperature for 1 h. Immobilon western chemiluminescent HRP substrate (Millipore, WBKLS0050) was used to visualize the membranes. ImageJ was used to quantify band intensities.

### RNA interference

Small interfering RNAs (siRNAs) for PCAF and a negative control (NC) were constructed by Gene Pharma (Shanghai, China). The sequences of all the siRNAs are listed in Table S3. Cells were transfected with the indicated siRNA or NC siRNA at a dose of 1 OD per 1.5 × 10^6^ cells with Lipofectamine RNAi MAX (Thermo). After 6 h, the medium was removed, and the cells were cultured under the indicated conditions for another 72 h. Then, the cells were collected for analysis of the knockdown efficiency by qRT‒PCR and western blotting.

### Plasmids

Expression plasmid constructs, including full-length pcDNA3.1(+)-Flag-PCAF (*Homo sapiens*), full-length pcDNA3.1(+)-HA-p65 (*H. sapiens*), full-length pcDNA3.1(+)-HA-p65K122/123R (*H. sapiens*), full-length pcDNA3.1(+)-HA-p65K218R (*H. sapiens*), full-length pcDNA3.1 (+)-HA-p65K221R (*H. sapiens*), and full-length pcDNA3.1(+)-HA-p65K310R, were all constructed by IGE Biotechnology. A Lipofectamine 3000 transfection kit (Invitrogen, L3000-015) was used for transfection according to the manufacturer’s instructions. Briefly, 293T cells were seeded in 6-well plates at a density of 3 × 10^5^ cells/well. Approximately 2.5 µg of plasmid was used per well along with 5 µl of Lipo3000 and 5 µl of P3000. The total amount of transfected plasmid in each well was equalized by adding empty pcDNA3.1(+)-vector.

### In vivo biodistribution of DSNPs

After the DBA1/J mouse CIA animal model was prepared, cy5.5-DSNPs were injected into the tail vein of wild-type or CIA mice at a dose of 10 mg/kg 21 days after booster immunization. The in vivo biodistribution was then observed over time using a Xenogen IVIS Spectrum system. The organs were dissected 48 h after systemic administration of the nanoparticles. Ex vivo fluorescence images were also obtained using the Xenogen IVIS Spectrum system. Fluorescence images were obtained using wavelengths of 680 nm (excitation) and 710 nm (emission).

### Chromatin immunoprecipitation (ChIP)

ChIP assays were performed using the EZ-Magna ChIP A/G assay kit (Millipore, 17-10086) according to the manufacturer’s instructions. Briefly, cells were crosslinked with 1% formaldehyde at room temperature for 10 min, and the collected cells were lysed to isolate the nuclei with nuclear lysis buffer containing a protease inhibitor cocktail. Then, sonication was used to shear the chromatin and generate 200–1,000-bp DNA fragments. Finally, the sheared chromatin was immunoprecipitated with primary antibodies against p65 (Cell Signaling Technology, 8242 S) and H3K9ac (Abcam, ab32129), normal rabbit IgG and anti-RNA polymerase II antibody (provided in the kit). After protein‒DNA crosslinking had been reversed, the precipitated DNA was purified and subsequently analyzed by qRT‒PCR. Data were normalized to the input control. The primers used for ChIP‒qPCR are listed in Supporting Information Table S4.

### RNA sequencing and data analysis

Human peripheral blood mononuclear cells (PBMCs) were isolated from the venous blood of 3 independent healthy donors. The detailed methods for obtaining monocytes from PBMCs have been described previously. To obtain M0 macrophages, monocytes were cultured with 10% FBS-containing 1640 medium supplemented with 25 ng/ml M-CSF (PeproTech, 300-25-10) at 37 °C for 5 days. To induce the formation of M1 macrophages, 1 mg/ml LPS (Sigma, L2880) and 30 ng/ml IFN-γ (Peprotech, 300-02-20) were added on the 6th day. Total RNA was extracted from 3 repeats of M0, 3 repeats of M1 without 20 µM garcinol and 3 repeats of M1 with 20 µM garcinol as described above. cDNA library construction and sequencing were performed by the Beijing Genomics Institute using the BGISEQ platform. Differential expression analysis was performed with the following parameters: fold change ≥ 1.5 and adjusted *P* value ≤ 0.05. Sequencing data analysis, including heatmap clustering, Venn diagram creation, gene ontology (GO) analysis, Kyoto Encyclopedia of Genes and Genomes (KEGG) analysis, and gene set enrichment analysis (GSEA), was performed using BGI Dr. Tom 2.0.

### Statistical analysis

Statistical analysis was performed using SPSS 18.0 software. All data are expressed as the mean ± SD of three independent experiments. Student’s t test and one-way analysis of variance followed by the Bonferroni test were performed for statistical analyses. Differences for which *P* < 0.05 were considered statistically significant.

### Electronic supplementary material

Below is the link to the electronic supplementary material.

Supplementary Material 1

Supplementary Material 2

Supplementary Material 3

Supplementary Material 4

Supplementary Material 5 Supplementary Material 5

Supplementary Material 6 Supplementary Material 6

Supplementary Material 7 Supplementary Material 7

Supplementary Material 8 Supplementary Material 8

Supplementary Material 9 Supplementary Material 9

## Data Availability

The raw/processed data required to reproduce these findings are available to download from https://www.ncbi.nlm.nih.gov/geo/. Data sets from RNA-seq have been deposited in the NCBI Gene Expression Omnibus under accession number GSE221012.
