# Correction: Targeting macrophage M1 polarization suppression through PCAF inhibition alleviates autoimmune arthritis via synergistic NF-κB and H3K9Ac blockade

**DOI:** 10.1186/s12951-023-02079-8

**Published:** 2023-09-20

**Authors:** Jinteng Li, Feng Ye, Xiaojun Xu, Peitao Xu, Peng Wang, Guan Zheng, Guiwen Ye, Wenhui Yu, Zepeng Su, Jiajie Lin, Yunshu Che, Zhidong Liu, Pei Feng, Qian Cao, Dateng Li, Zhongyu Xie, Yanfeng Wu, Huiyong Shen

**Affiliations:** 1 Department of Orthopedics, The Eighth Affiliated Hospital of Sun Yat-sen University, Shenzhen, 518003 PR China; 2 Center for Biotherapy, The Eighth Affiliated Hospital of Sun Yat-sen University, Shenzhen, 518003 PR China; 3 121 Westmoreland Ave, White Plains, 10606 NY USA; 4 Shenzhen Key Laboratory of Ankylosing Spondylitis, Shenzhen, 518003 PR China; 5 Guangdong Orthopedic Clinical Research Center, Shenzhen, 518003 PR China

**Correction: Journal of Nanobiotechnology (2023) 21:280**

10.1186/s12951-023-02012-z

In this article, Wenhui Yu was incorrectly denoted as the corresponding author but it should have been Zhongyu Xie, Yanfeng Wu and Huiyong Shen.

The original article [1] has been corrected.

## References

[1] Li J, Ye F, Xu X, et al. Targeting macrophage M1 polarization suppression through PCAF inhibition alleviates autoimmune arthritis via synergistic NF-κB and H3K9Ac blockade. J Nanobiotechnol 2023;21:280. 10.1186/s12951-023-02012-z 10.1186/s12951-023-02012-z PMC10439630 37598147

