# Supplementary material for: Targeting macrophage M1 polarization suppression through PCAF inhibition alleviates autoimmune arthritis via synergistic NF-κB and H3K9Ac blockade
Source: J Nanobiotechnology. 2023 Aug 19;21:280. doi: 10.1186/s12951-023-02012-z (PMC10439630; doi:10.1186/s12951-023-02012-z)
Supplement: Supplementary file 1 — Supplementary Material 1 [file 12951_2023_2012_MOESM1_ESM.docx]

**Supplemental Table S1: The source and purity of the reagents.**

| Regeant | Purity | Source |
| --- | --- | --- |
| Dextran sulfate | ≥99.9% | Maclin, Shanghai, China |
| 4-Nitrophenyl Chloroformate | 90% | Maclin, Shanghai, China |
| 5β-cholic acid | ≥99% | Sigma |
| Pyridine | AR | Sinopharm chemical reagent, China |
| DMSO | AR | Sinopharm chemical reagent, China |
| Ether | AR | Sinopharm chemical reagent, China |
| Ethylenediamine | AR | Sinopharm chemical reagent, China |
| Methanol | AR | Sinopharm chemical reagent, China |
| Concentrated hydrochlo | - | Sinopharm chemical reagent, China |
