# Supplementary material for: Targeting macrophage M1 polarization suppression through PCAF inhibition alleviates autoimmune arthritis via synergistic NF-κB and H3K9Ac blockade
Source: J Nanobiotechnology. 2023 Aug 19;21:280. doi: 10.1186/s12951-023-02012-z (PMC10439630; doi:10.1186/s12951-023-02012-z)
Supplement: Supplementary file 2 — Supplementary Material 2 [file 12951_2023_2012_MOESM2_ESM.docx]

**Supplemental Table S2. Primers used for qRT-PCR.**

| **Gene** |  | **Primer sequence (5’-3’)** |
| --- | --- | --- |
| *GAPDH* | Forward | TGTGGGCATCAATGGATTTGG |
|  | Reverse | ACACCATGTATTCCGGGTCAAT |
| *CBP* | Forward | CAACCCCAAAAGAGCCAAACT |
|  | Reverse | CCTCGTAGAAGCTCCGACAGT |
| *PCAF* | Forward | CGAATCGCCGTGAAGAAAGC |
|  | Reverse | CTTGCAGGCGGAGTACACT |
| *P300* | Forward | AGCCAAGCGGCCTAAACTC |
|  | Reverse | TCACCACCATTGGTTAGTCCC |
| GCN5 | Forward | GCAAGGCCAATGAAACCTGTA |
|  | Reverse | TCCAAGTGGGATACGTGGTCA |
| *TNF-α* | Forward | GAGGCCAAGCCCTGGTATG |
|  | Reverse | CGGGCCGATTGATCTCAGC |
| *IL-6* | Forward | ACTCACCTCTTCAGAACGAATTG |
|  | Reverse | CCATCTTTGGAAGGTTCAGGTTG |
| *IL-1β* | Forward | ATGATGGCTTATTACAGTGGCAA |
|  | Reverse | GTCGGAGATTCGTAGCTGGA |
