# Supplementary material for: Targeting macrophage M1 polarization suppression through PCAF inhibition alleviates autoimmune arthritis via synergistic NF-κB and H3K9Ac blockade
Source: J Nanobiotechnology. 2023 Aug 19;21:280. doi: 10.1186/s12951-023-02012-z (PMC10439630; doi:10.1186/s12951-023-02012-z)
Supplement: Supplementary file 3 — Supplementary Material 3 [file 12951_2023_2012_MOESM3_ESM.docx]

**Supplemental Table S3. Sequences of siRNAs used for gene knockdown.**

| **siRNA** | **Sequence (5’-3’)** | |
| --- | --- | --- |
| *NC* | Sense | UUCUCCGAACGUGUCACGUTT |
|  | Antisense | ACGUGACACGUUCGGAGAATT |
| *PCAF S1* | Sense | GCAGAUACCAAACAAGUUUTT |
|  | Antisense | AAACUUGUUUGGUAUCUGCTT |
| *PCAF S2* | Sense | GCGACAACUCCUGGAACAATT |
|  | Antisense | UUGUUCCAGGAGUUGUCGCTT |
|  |  |  |
|  |  |  |
