# Supplementary material for: Targeting macrophage M1 polarization suppression through PCAF inhibition alleviates autoimmune arthritis via synergistic NF-κB and H3K9Ac blockade
Source: J Nanobiotechnology. 2023 Aug 19;21:280. doi: 10.1186/s12951-023-02012-z (PMC10439630; doi:10.1186/s12951-023-02012-z)
Supplement: Supplementary file 4 — Supplementary Material 4 [file 12951_2023_2012_MOESM4_ESM.docx]

**Supplemental Table S4.** **Primers used for ChIP-qPCR.**

| **Gene** |  | **Primer sequence (5’-3’)** |
| --- | --- | --- |
| *TNF-α* | Forward | TGGGGGTAGGGTTAGTACCG |
|  | Reverse | CTACAGGCTTGTCACTCGGG |
| *IL-6* | Forward | GCTCCCTACACACATGCCTT |
|  | Reverse | CCTTCCCTGTGCATGGTGAT |
| *IL-1β* | Forward | ACTACCAGTCCTGACTCCCT |
|  | Reverse | GCCACCGAAGACTATCCTCC |
|  |  |  |
|  |  |  |
