# Supplementary figures and images for: Targeting macrophage M1 polarization suppression through PCAF inhibition alleviates autoimmune arthritis via synergistic NF-κB and H3K9Ac blockade
Source: J Nanobiotechnology. 2023 Aug 19;21:280. doi: 10.1186/s12951-023-02012-z (PMC10439630; doi:10.1186/s12951-023-02012-z)

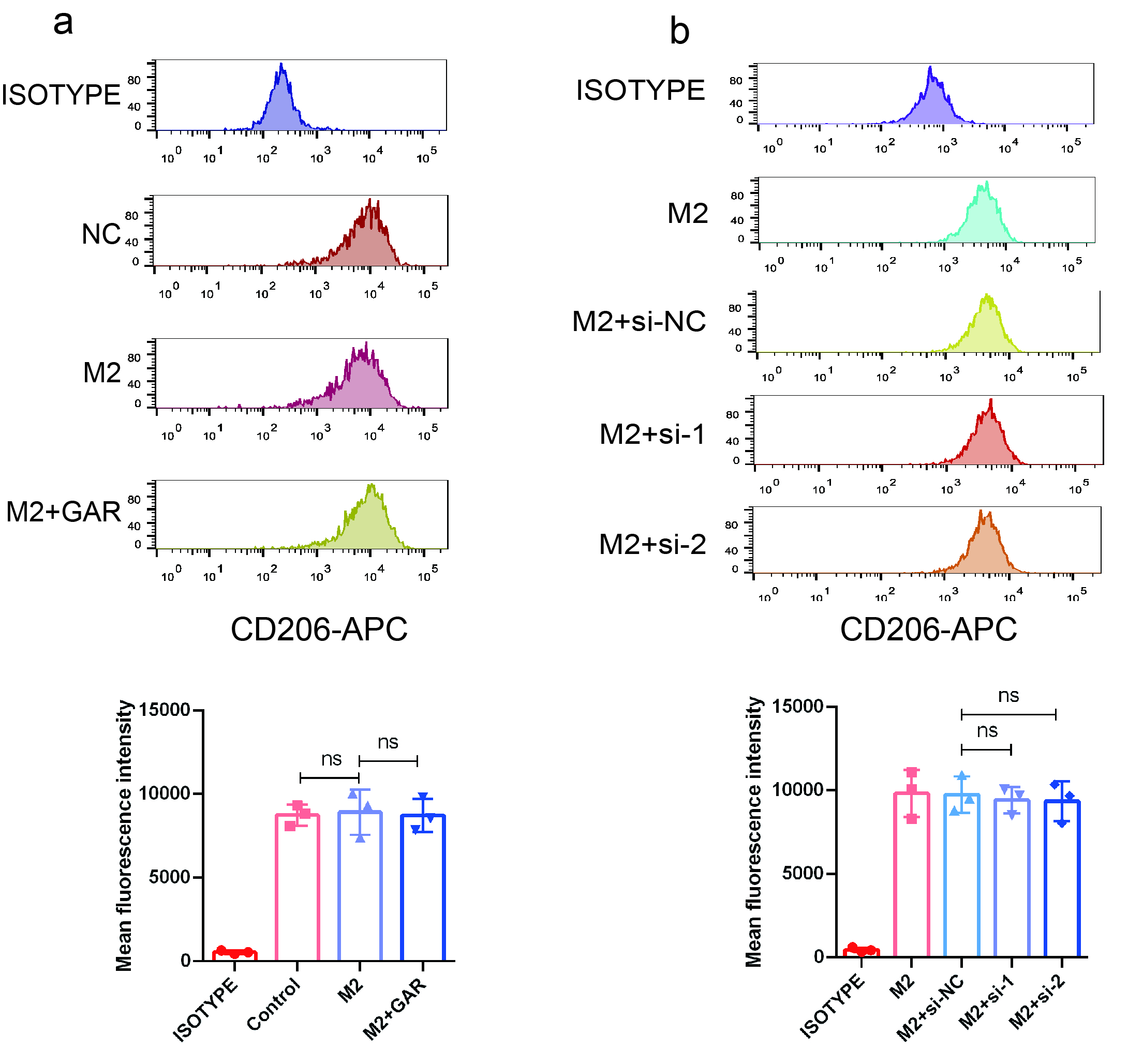

Supplement: Supplementary file 5 — Supplementary Material 5 [file 12951_2023_2012_MOESM5_ESM.jpg]

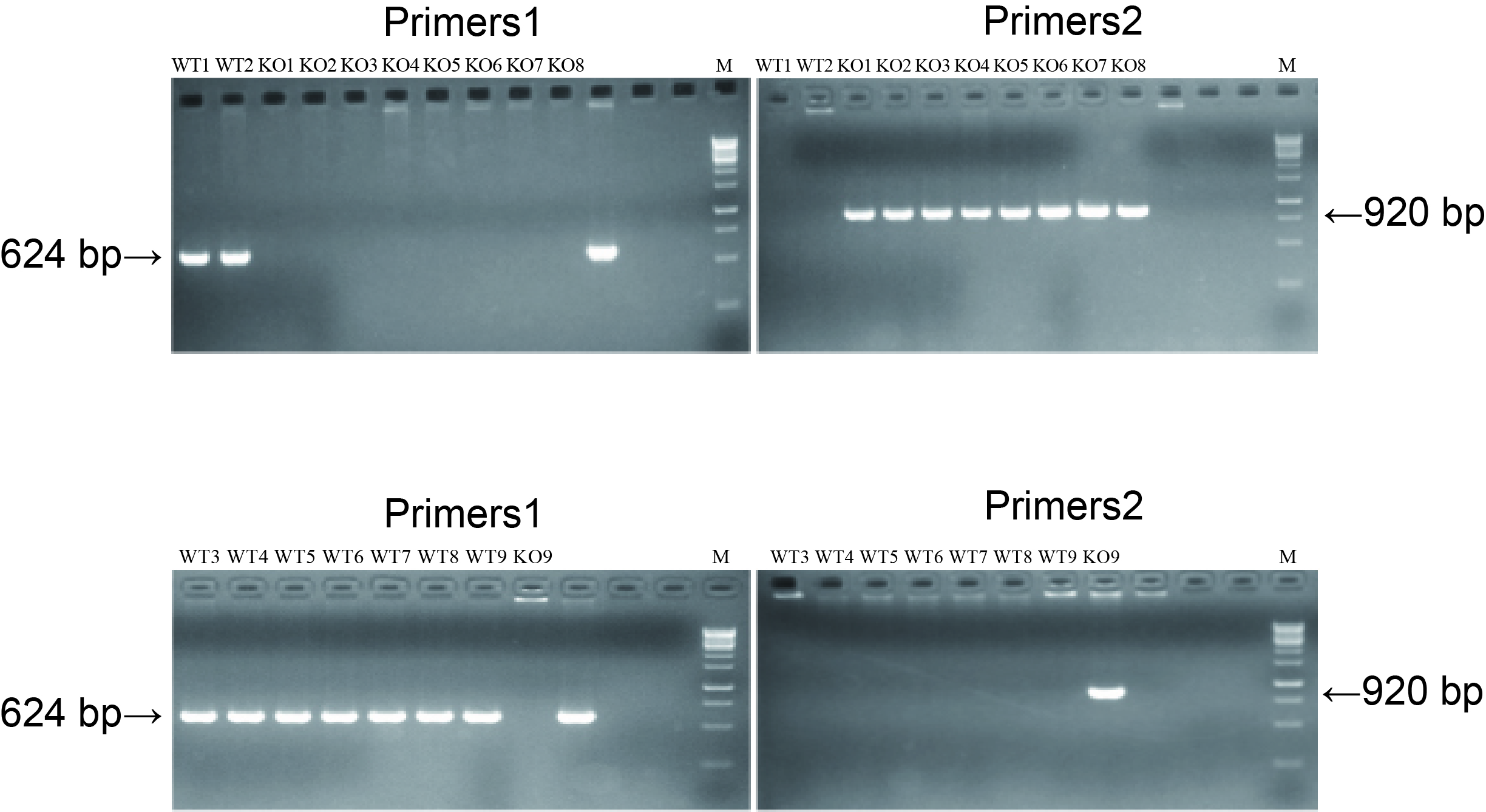

Supplement: Supplementary file 6 — Supplementary Material 6 [file 12951_2023_2012_MOESM6_ESM.jpg]

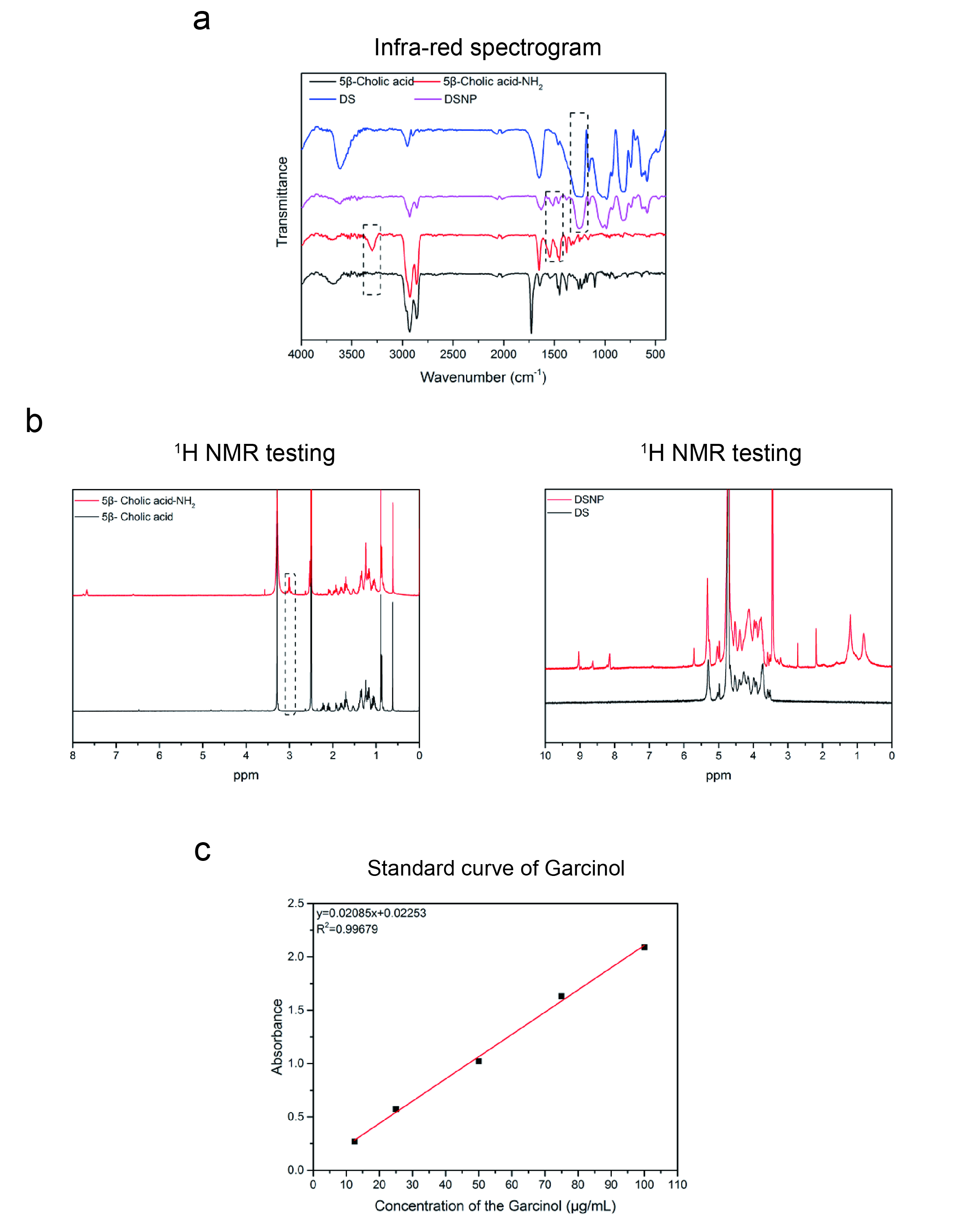

Supplement: Supplementary file 7 — Supplementary Material 7 [file 12951_2023_2012_MOESM7_ESM.jpg]

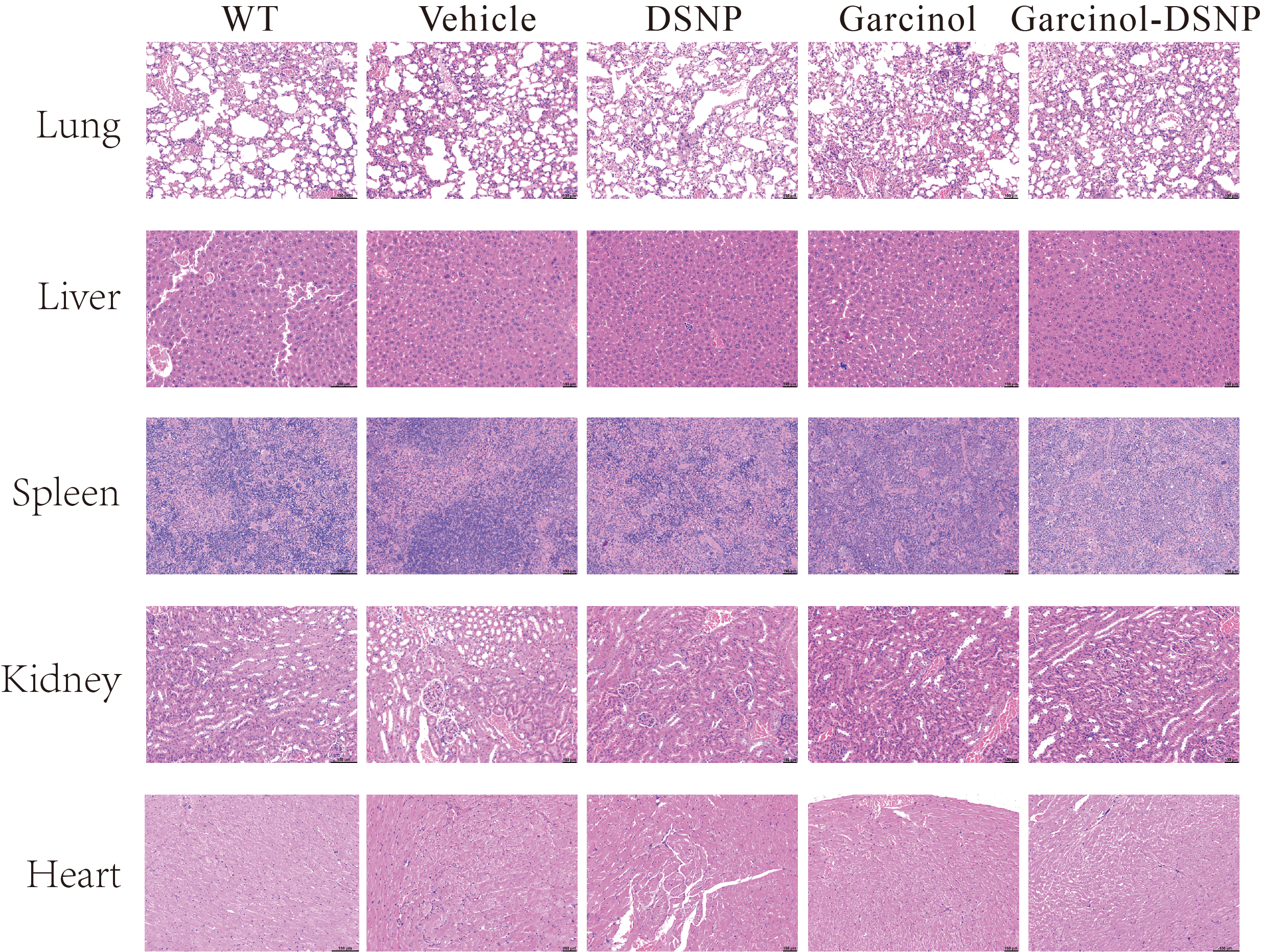

Supplement: Supplementary file 8 — Supplementary Material 8 [file 12951_2023_2012_MOESM8_ESM.jpg]

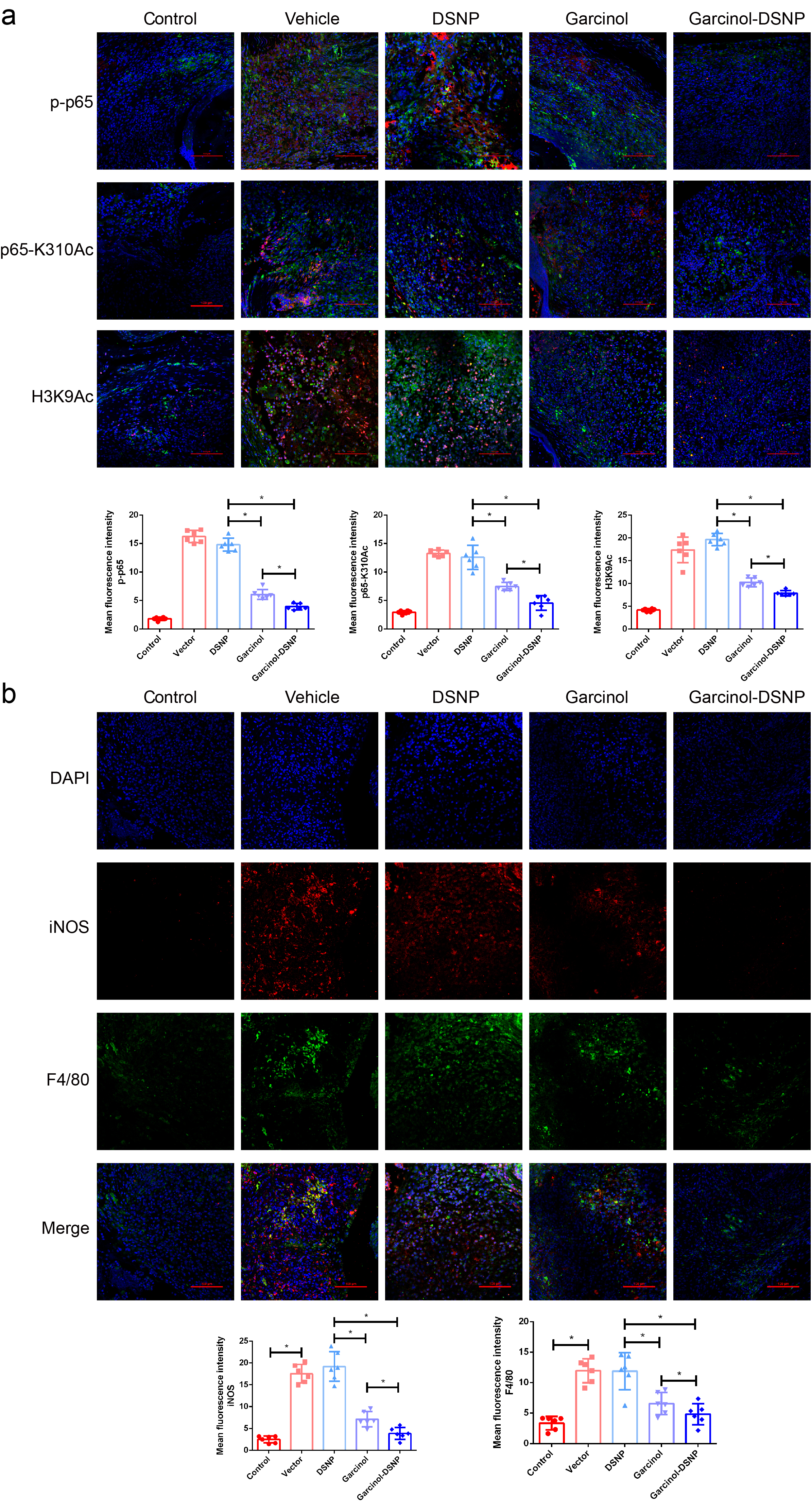

Supplement: Supplementary file 9 — Supplementary Material 9 [file 12951_2023_2012_MOESM9_ESM.jpg]
